# Antimicrobial Activity of the Marine Alkaloids, Clathrodin and Oroidin, and Their Synthetic Analogues

**DOI:** 10.3390/md12020940

**Published:** 2014-02-14

**Authors:** Nace Zidar, Sofia Montalvão, Žiga Hodnik, Dorota A. Nawrot, Aleš Žula, Janez Ilaš, Danijel Kikelj, Päivi Tammela, Lucija Peterlin Mašič

**Affiliations:** 1 Faculty of Pharmacy, University of Ljubljana, Aškerčeva 7, Ljubljana 1000, Slovenia; E-Mails: nace.zidar@ffa.uni-lj.si (N.Z.); ziga.hodnik@ffa.uni-lj.si (Ž.H.); ales.zula@ffa.uni-lj.si (A.Ž.); janez.ilas@ffa.uni-lj.si (J.I.); danijel.kikelj@ffa.uni-lj.si (D.K.); 2 Centre for Drug Research, Faculty of Pharmacy, University of Helsinki, P.O. Box 56 (Viikinkaari 5 E), Helsinki FI-00014, Finland; E-Mails: sofia.montalvao@helsinki.fi (S.M.); dorota.nawrot@helsinki.fi (D.A.N.); 3 Division of Pharmaceutical Biology, Faculty of Pharmacy, University of Helsinki, P.O. Box 56 (Viikinkaari 5 E), Helsinki FI-00014, Finland

**Keywords:** marine alkaloid, *Agelas*, antimicrobial, antibacterial, antifungal, pyrrole-2-aminoimidazole, oroidin, clathrodin

## Abstract

Marine organisms produce secondary metabolites that may be valuable for the development of novel drug leads as such and can also provide structural scaffolds for the design and synthesis of novel bioactive compounds. The marine alkaloids, clathrodin and oroidin, which were originally isolated from sponges of the genus, *Agelas*, were prepared and evaluated for their antimicrobial activity against three bacterial strains (*Enterococcus faecalis*, *Staphylococcus* *aureus* and *Escherichia* *coli*) and one fungal strain (*Candida albicans*), and oroidin was found to possess promising Gram-positive antibacterial activity. Using oroidin as a scaffold, 34 new analogues were designed, prepared and screened for their antimicrobial properties. Of these compounds, 12 exhibited >80% inhibition of the growth of at least one microorganism at a concentration of 50 µM. The most active derivative was found to be 4-phenyl-2-aminoimidazole **6h**, which exhibited MIC_90_ (minimum inhibitory concentration) values of 12.5 µM against the Gram-positive bacteria and 50 µM against *E. coli*. The selectivity index between *S. aureus* and mammalian cells, which is important to consider in the evaluation of a compound’s potential as an antimicrobial lead, was found to be 2.9 for compound **6h**.

## 1. Introduction

Marine natural products constitute a vital pool of biologically active compounds that are amenable to drug discovery. The marine ecosystem is a rich source of chemically and functionally diverse molecules that function in their native environment as offensive weapons to capture pray or for protection against predators [1]. Sponges, for example, have been shown to produce secondary metabolites with highly promising antimicrobial activities [2]. Marine organisms produce some of the most potent bioactive compounds discovered to date. However, because the concentrations of these compounds are usually very low, natural sources are unlikely to provide sufficient material for isolation and detailed biological evaluation, and chemical synthesis is often necessary to investigate their mode of action and their biological implications. This option is often hampered by the fact that natural compounds are also known for their high molecular weight, large number of chiral centers and complex 3D structures, which limit their synthetic availability and make them non-drug-like.

**Figure 1:**
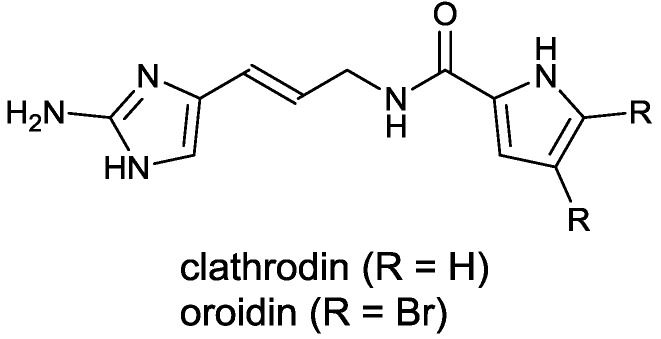
Structures of the marine alkaloids, clathrodin and oroidin.

Alkaloids initially isolated from the sponges of the genus, *Agelas*, e.g., clathrodin and oroidin (Figure 1), belong to the pyrrole-2-aminoimidazole structural class of secondary metabolites and exhibit intriguing structural complexity and increasingly studied biological activities; thus, these compounds are attracting the attention of a growing number of researchers from numerous disciplines worldwide [3,4]. Apart from the genus, *Agelas*, several other genera of sponges, e.g., *Hymeniacidon*, *Cymbaxinella* and *Axinella*, have also been identified to produce these alkaloids [4,5]. Compared with many other pyrrole-2-aminoimidazoles, the structures of the oroidin class of alkaloids (the key precursor for this group) are relatively simple and are thus suitable candidates for optimization using established medicinal chemistry strategies. Because of its relatively low molecular mass and simple structure, oroidin offers several possibilities for chemical optimization through the introduction of additional side chains or functional groups.

Infectious diseases remain one of the leading global causes of death. The widespread occurrence of bacterial strains resistant to the currently used antimicrobials represents a significant health threat; therefore, novel structural classes of antimicrobials with novel mechanisms of action are urgently needed [6,7,8,9,10]. Marine alkaloids from *Agelas* sponges and their synthetic analogues have been extensively studied as inhibitors of bacterial biofilm formation [11,12,13,14,15,16,17,18,19,20] and as antibacterial [21,22,23], antifungal [24] and antiprotozoal [25,26] agents. Some mechanisms of antimicrobial and antibiofilm action have been proposed, and these include disruption of the bacterial cell membrane [27], targeting the response regulator protein, BfmR [11], and inhibition of enoyl reductases [26].

In our study, we first prepared two natural marine alkaloids, namely, clathrodin and oroidin, and evaluated their antimicrobial activity against a panel of laboratory strains of known pathogens, including Gram-positive (*Enterococcus faecalis* and *Staphylococcus* *aureus*) and Gram-negative bacteria (*Escherichia coli*) and fungi (*Candida albicans*). Our initial results revealed that clathrodin possessed almost no antimicrobial activity, but its dibromo analogue, oroidin, showed promising inhibition of the growth of the Gram-positive bacteria, *S.* *aureus* and *E. faecalis*. These results indicate that larger, lipophilic moieties, rather than the pyrrole ring found in the clathrodin molecule, are required for good antibacterial activity. The primary screen results stimulated us to prepare a series of oroidin analogues with pyrrol-replacing groups, such as indole and substituted indole rings. Using different substituents on the indole ring, we investigated the chemical properties required for biological activity. To further explore the structure-activity relationships, we also designed a series of conformationally restricted oroidin analogues. In addition to their antimicrobial evaluation, the *in vitro* cytotoxicity of these compounds on mammalian cells was determined to further assess the selectivity of the active compounds toward different prokaryotic and eukaryotic cells.

## 2. Results and Discussion

### 2.1. Design

**Figure 2:**
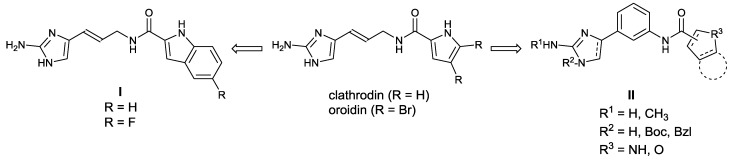
The design of 4-(3-aminoprop-1-en-1-yl)-2-aminoimidazoles (**I**) and 4-phenyl-2-aminoimidazoles (**II**) as clathrodin and oroidin analogues with potential antimicrobial activity.

**Scheme 1:**
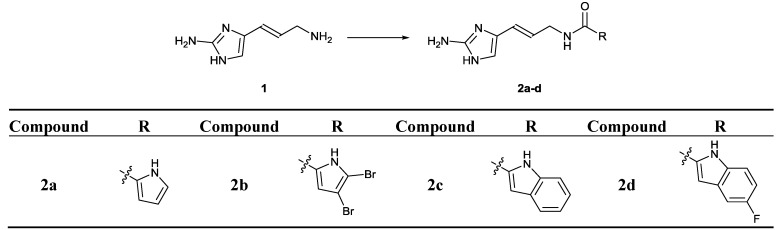
Synthesis of clathrodin (**2a**), oroidin (**2b**) and their indole (**2c**) and 5-fluoro-indole (**2d**) analogues. Reagents and conditions: corresponding carboxylic acid, TBTU, NMM, DMF, rt, 6 h.

**Scheme 2:**
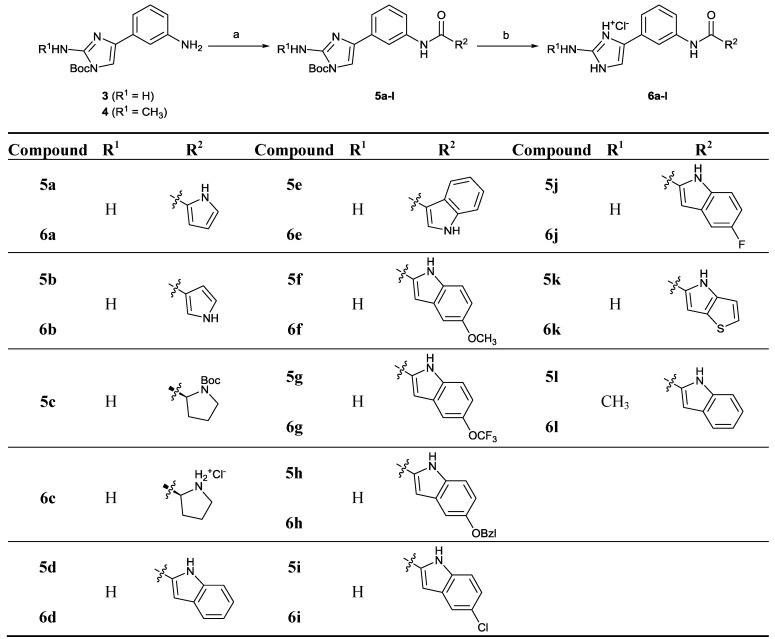
Synthesis of 4-phenyl-2-aminoimidazoles **5a**–**k** and **6a**–**k** and 4-phenyl-2-(*N*-methylamino)-imidazoles **5l** and **6l**. Reagents and conditions: (**a**) Corresponding carboxylic acid, TBTU, NMM, CH_2_Cl_2_, 35 °C, 24 h; (**b**) HCl_(g)_, THF/EtOH, rt, 5 h.

**Scheme 3:**
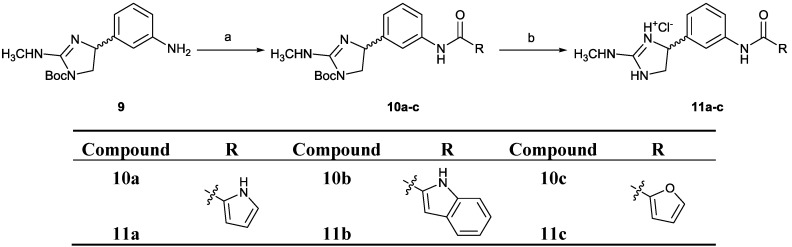
Synthesis of 4-phenyl-4,5-dihydro-(*N*-methylamino)-imidazoles **10a**–**c** and **11a**–**c**. Reagents and conditions: (**a**) Corresponding carboxylic acid, TBTU, NMM, CH_2_Cl_2_, 35 °C, 24 h; (**b**) HCl_(g)_, EtOH, rt, 5 h.

**Scheme 4:**
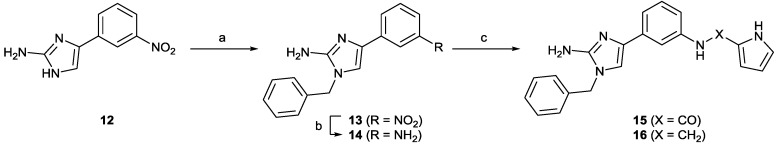
Synthesis of 1-benzyl-4-phenyl-2-aminoimidazoles **13** and **14**. Reagents and conditions: (**a**) Benzyl bromide, K_2_CO_3_, CH_3_CN, 50 °C, 14 h; (**b**) H_2_/Pd-C, THF, rt, 5 h; (**c**) pyrrole-2-carboxylic acid, TBTU, Et_3_N, CH_2_Cl_2_, rt, 18 h (for the synthesis of **15**); pyrrole-2-carbaldehyde, NaBH(OAc)_3_, CH_3_COOH, CH_2_Cl_2_, rt, 13 h (for the synthesis of **16**).

We designed two main classes of oroidin analogues, * i.e.*, 4-(3-aminoprop-1-en-1-yl)-2-aminoimidazoles **I** and 4-phenyl-2-aminoimidazoles **II** (Figure 2). The structures of the class **I** compounds are closely related to natural alkaloids and were obtained through the replacement of the pyrrole or 2,3-dibromo-pyrrole rings with indole and 5-fluoro-indole moieties (Scheme 1, compounds **2c** and **2d**). With respect to the class **II** series, a set of 34 analogues was designed and prepared by introducing a phenyl ring into position 4 of the 2-aminoimidazole ring. In that way, a conformational constraint was introduced into the molecule to limit the flexibility of the compounds without altering the length of the molecule compared with the natural alkaloids. In addition to compounds with a pyrrol-2-yl substituent, which is present in clathrodin, analogues with pyrrol-3-yl, (*R*)-pyrrolidin-2-yl, indol-2-yl, indol-3-yl, thieno[3,2-*b*]pyrrol-5-yl and furan-2-yl substituents were prepared. With the introduction of these groups, we wanted to explore their optimal size and the hydrophobic/hydrophilic properties required for biological activity. In that respect, different 5-substituted indole rings were also studied. To assess the importance of the free primary amino group of the 2-aminoimidazole moiety, a set of 4-phenyl-(*N*-methylamino)-imidazoles, namely **5l** and **6l** (Scheme 2) and **10a**–**c** and **11a**–**c** (Scheme 3), was synthesized. Furthermore, the effects of different substituents on the imidazole *N*-1 nitrogen, such as Boc (*tert*-butyl-oxy-carbonyl) and benzyl (Scheme 4, compounds **13**–**16**), were studied. Finally, a set of 4,5-dihydro-2-aminoimidazoles (Scheme 3, compounds **10a**–**c** and **11a**–**c**) with a reduced imidazole C=C bond was prepared and evaluated. With that modification, the effects of aromaticity and planarity of the imidazole ring on the biological activity were assessed.

### 2.2. Chemistry

Clathrodin (**2a**) and its indole (**2c**) and 5-fluoro-indole (**2d**) analogues were prepared by coupling amine **1** [28] and the appropriate carboxylic acid (pyrrole-2-carboxylic acid, 4,5-dibromo-pyrrole-2-carboxylic acid, indole-2-carboxylic acid or 5-fluoro-indole-2-carboxylic acid), as depicted in Scheme 1.

The 4-phenyl-2-aminoimidazoles **5a**–**k** and **6a**–**k** and the 4-phenyl-2-(*N*-methylamino)-imidazoles **5l** and **6l **were synthesized according to Scheme 2. First, *N*-Boc-protected derivatives **5a**–**l** were prepared in a TBTU-promoted coupling reaction between *tert*-butyl 2-amino-4-(3-aminophenyl)-1*H*-imidazole-1-carboxylate (**3**) or *tert*-butyl 4-(3-aminophenyl)-2-(methylamino)-1*H*-imidazole-1-carboxylate (**4**) and various carboxylic acids (pyrrole-2-carboxylic acid, pyrrole-3-carboxylic acid, *N*-Boc-d-proline, indole-2-carboxylic acid, indole-3-carboxylic acid, 5-substituted indole-2-carboxylic acids or 4*H*-thieno[3,2-*b*]pyrrole-5-carboxylic acid). Next, the *N*-Boc protecting groups of **5a**–**l** were removed with gaseous hydrochloric acid to obtain the target compounds **6a**–**l**. The detailed procedures for the syntheses of *tert*-butyl 2-amino-4-(3-aminophenyl)1*H*-imidazole-1-carboxylate (**3**) and *tert*-butyl 4-(3-aminophenyl)-2-(methylamino)-1*H*-imidazole-1-carboxylate (**4**) are described elsewhere [29].

**Scheme 5:**
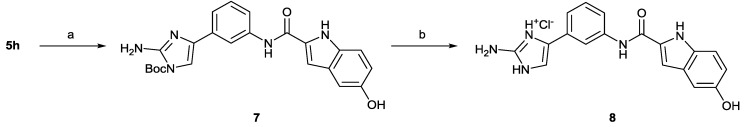
Synthesis of 4-phenyl-2-aminoimidazoles **7** and **8**. Reagents and conditions: (**a**) H_2_/Pd-C, THF/MeOH, rt, 10 h; (**b**) HCl_(g)_, THF/EtOH, rt, 5 h.

Compound **8**, which contains a 5-hydroxyl substituent on the indole ring, was prepared from the 5-benzyloxy-indol derivative **5h** through the two-step procedure depicted in Scheme 5. After a palladium-catalyzed hydrogenation to remove the *O*-benzyl group, the obtained compound **7** was converted into the target 4-phenyl-2-aminoimidazole **8** upon cleavage of the Boc protecting group with gaseous hydrochloric acid.

The 4-phenyl-4,5-dihydro-(*N*-methylamino)-imidazoles **11a**–**c** were prepared using the above-described procedure for the syntheses of compounds **6a**–**l **(Scheme 3). The TBTU-promoted coupling reactions between amine **9** and different carboxylic acids (pyrrole-2-carboxylic acid, indole-2-carboxylic acid or furan-2-carboxylic acid) yielded compounds **10a**–**c**, which were converted into **11a**–**c** upon cleavage of the *N*-Boc protecting group with gaseous hydrochloric acid. The synthesis of *tert*-butyl 4-(3-aminophenyl)-2-(methylamino)-4,5-dihydro-1*H*-imidazole-1-carboxylate (**9**) is reported elsewhere [29].

For the preparation of compounds **15** and **16**, which contain a benzyl group on *N*-1 of the imidazole ring (Scheme 4), 4-(3-nitrophenyl)-2-aminoimidazole (**12**) was first reacted with benzyl bromide in the presence of potassium carbonate to obtain the 1-benzylated derivative **13**. The nitro group of **13** was then reduced through catalytic hydrogenation, and the obtained amine **14** was coupled with pyrrole-2-carboxylic acid to afford the target compound **15**. 1-Benzyl-4-phenyl-2-aminoimidazole **16**, an analogue of **15** with a reduced amide bond, was obtained using sodium triacetoxyborohydride to achieve the reductive amination of **14** with pyrrole-2-carbaldehyde.

### 2.3. Biological Evaluation

**Figure 3:**
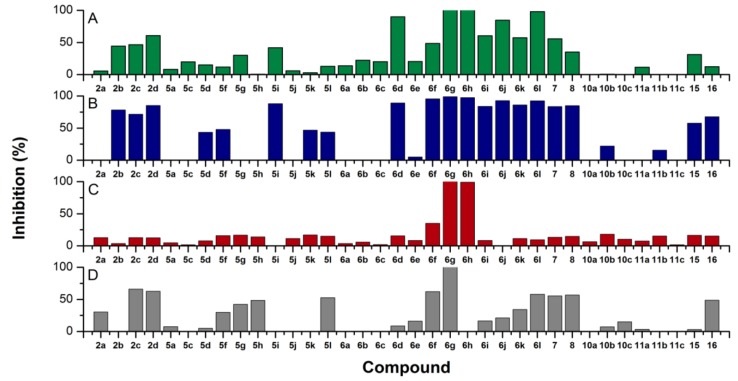
Primary antimicrobial screening results for clathrodin (**2a**), oroidin (**2b**) and their analogues at a concentration of 50 µM against: (**A**) *Enterococcus faecalis* (ATCC 29212); (**B**) *Staphylococcus aureus* (ATCC 25923); (**C**) *Escherichia coli* (ATCC 25922); and (**D**) *Candida albicans* (ATCC 90028). The results are based on the activity measured after 24 h (bacteria) or 48 h (*C. albicans*) of incubation (*n* = 3). Ciprofloxacin was used as a reference antibiotic in the antibacterial assays; MIC_90_ (minimum inhibitory concentration) values for *E. faecalis*, *S. aureus* and *E. coli* were 3, 1.5 and 0.048 µM (1, 0.5 and 0.016 µg/mL), respectively. And amphotericin B was used as a reference in the antifungal assay (MIC_90_ = 0.5 µM (0.5 µg/mL).

The compounds belonging to both structural classes, *i.e.*, 4-(3-aminoprop-1-en-1-yl)-2-aminoimidazoles **I** (**2a**–**d**) and 4-phenyl-2-aminoimidazoles **II** (**5a**–**c**, **5d**, **5f**–**l**, **6a**–**l**, **7**, **8**, **10a**–**c**, **11a**–**c**, **15** and **16**) were evaluated for their antimicrobial activity against three bacterial strains (Gram-positive *Enterococcus faecalis* ATCC 29212 and *Staphylococcus aureus* ATCC 25923 and Gram-negative *Escherichia coli* ATCC 25922) and one fungal strain (*Candida albicans* ATCC 90028). The primary screening results at a concentration of 50 µM are presented in Figure 3. The antimicrobial screening assays were performed using broth microdilution method, as detailed in the Experimental Section. The minimum inhibitory concentrations (MIC_50_, MIC_90_) were further determined for those compounds that showed >80% inhibition of growth in the primary screen (Table 1). In addition, the selected compounds were also tested for mammalian cell cytotoxicity to determine the selectivity indices (SI) for their antimicrobial effects (Table 1 and Table 2).

The parent compound, clathrodin (**2a**), exhibited activities below the hit threshold (>80% inhibition of growth at a concentration of 50 μM) against all of the microbial strains tested. Interestingly, its dibromo-pyrrole analogue, oroidin (**2b**), showed noticeably higher antibacterial activity against the Gram-positive bacteria, *S. aureus* (>90% inhibition of growth) and *E. faecalis* (approximately 50% inhibition of growth), but was also inactive against *C. albicans* and the Gram-negative bacteria, *E. coli*. Based on these results, a set of oroidin analogues was designed and prepared, *i.e.*, the dibromo-pyrrole ring was substituted with other groups, such as indole and substituted indole rings with similar spatial and hydrophobic/hydrophilic properties to dibromo-pyrrole. Based on the primary screening results, the hit rates (>80% inhibition of growth) of all 36 tested compounds against *S. aureus*, *E. faecalis*, *E. coli* and *C. albicans* were 33, 14, six and 3%, respectively (Figure 3). Twelve compounds were active against *S. aureus*, and five of these were also active against the other Gram-positive bacterium*, E. faecalis*. The majority of the active compounds belonged to the 4-phenyl-2-aminoimidazole structural class, which is presented in Scheme 2 and Scheme 5. In general, the most active compounds were analogues containing an indol-2-yl, 5-substituted indol-2yl or thieno[3,2-*b*]pyrrol-5-yl group in the eastern part of the molecule and also possessing unsubstituted imidazole *N*-1 nitrogen (**6d**, **6f**–**l**). Interestingly, compound **6e**, which contains an indol-3-yl substituent, was inactive. Those compounds with smaller substituents in the eastern part, such as pyrrol-2-yl (**5a**, **6a**), pyrrol-3-yl (**6b**) and (*R*)-pyrrolidin-2-yl (**5c**, **6c**) and those compounds with Boc substituents on the imidazole *N*-1 nitrogen (**5a**, **5c**–**l**, **7** and **10a**–**c**), were less active. The only active compound containing a Boc substituent on the imidazole *N*-1 was compound **7**. In general, compounds with Boc substituents on the imidazole *N*-1 nitrogen (**5a**, **5c**–**l**, **7** and **10a**–**c**) were less active than the products with free amino groups (**6a**, **6c**–**l**, **8** and **11a**–**c**); therefore, some Boc analogues, like compound **5b**, were not tested, as no increase in the biological activity could be anticipated. According to these findings, the unsubstituted imidazole *N*-1 nitrogen plays an important role in enhancing the biological activity, possibly by forming direct or indirect interactions with the target. The most promising results were obtained for the 4-phenyl-2-aminoimidazoles, **6g **and **6h**, containing 5-*O*-substituted indole rings. Interestingly, compound **6g** showed full inhibition of growth of all four microbes tested, whereas compound **6h**, which contains a large, lipophilic 5-benzyloxy substituent on the indole ring, was active against all bacterial strains tested, but showed no activity against *C. albicans*. Compounds structurally similar to **6g** have been identified as inhibitors of the biofilm formation in Gram-negative bacteria [18]. The 4-phenyl-4,5-dihydro-(*N*-methylamino)-imidazoles, **10a**–**c** and **11a**–**c**, which contain a reduced imidazole C=C bond, were not active, which indicates the importance of the aromaticity and planarity of the imidazole ring for antimicrobial activity.

**Table 1:** MIC_90_ and MIC_50_ values and the selectivity indices (SI) used to evaluate the antimicrobial activities of oroidin (**2b**) and selected oroidin analogues. The SI values were calculated as the ratio between the antimicrobial MIC_50_ and the Huh-7 cytotoxicity IC_50_ values.

Cpd	*Enterococcus faecalis* (ATCC 29212)			*Staphylococcus aureus* (ATCC 25923)			*Escherichia coli* (ATCC 25922)			*Candida albicans* (ATCC 90028)		
	MIC_90_ (µM)	MIC_50_ (µM)	SI	MIC_90_ (µM)	MIC_50_ (µM)	SI	MIC_90_ (µM)	MIC_50_ (µM)	SI	MIC_90_ (µM)	MIC_50_ (µM)	SI
**2b**				100	30.6	>3.3						
**5i**				75	11.7	>8.6						
**6d**	50	34.0	1.2	50	29.4	1.4						
**6f**				50	11.9	2.7						
**6g**	25	11.4	2.7	25	9.0	3.5	25	17.7	1.8	50	30.5	1.0
**6h**	12.5	8.0	2.7	12.5	7.3	2.9	50	30.4	0.7			
**6i**				50	7.2	3.0						
**6j**	100	27.3	0.7	50	22.1	0.9						
**6k**				100	27.1	2.0						
**6l**	100	54.2	1.0	75	10.8	4.9						
**7**				100	11.6	8.1						
**8**				100	40.5	>2.5						

**Table 2:** Mammalian cell cytotoxicity of oroidin (**2b**) and selected oroidin analogues that showed >80% inhibition in the primary antimicrobial screening. The cytotoxicity IC_50_ values against a hepatocellular carcinoma cell line (Huh-7) were determined by an ATP assay after 24 h of exposure to the compound. The results are averages from two independent dose-response experiments.

Compound	Cytotoxicity
	IC_50_ (µM)
**2b**	>100 *
**5i**	>100 *
**6d**	42.3
**6f**	32.2
**6g**	31.0
**6h**	21.2
**6i**	21.7
**6j**	19.8
**6k**	53.6
**6l**	52.3
**7**	94.0
**8**	>100 *

* = highest concentration tested.

The dose-response experiments conducted using the active compounds confirmed the primary screening results and demonstrated the potency differences between the analogues (Table 1). The lowest MIC_90_ values were obtained for derivative **6h** with MIC_90_ values of 12.5 µM (5.7 µg/mL) against the Gram-positive bacteria and 50 µM against *E. coli*. The MIC_90_ values of the 5-trifluoromethoxy-indole derivative **6g** were 25 µM (10.9 µg/mL) against all of the bacteria and 50 µM against *C. albicans*. For all the other derivatives, the MIC_90_s were ≥50 µM. For comparison, antibiotics currently used for treating *Staphylococcus* infections show MIC_90_ values in *in vitro* conditions typically in the range of 0.1–8 µg/mL [30]. However, the MIC value is not the only factor to be considered; the significance of the findings may also depend, for example, on the structural novelty and selectivity profile of the compound. Thus, in addition to the antimicrobial activity, the cellular impacts of the most promising compounds on a hepatocyte cell line, Huh-7, after 24 h of exposure were also evaluated to measure the selectivity of the effects between prokaryotic and eukaryotic cells. Clearly, most of the antimicrobially active derivatives showed a broad-spectrum effect, targeting both prokaryotic and eukaryotic cells (Table 2); however, in general, higher IC_50_ values were obtained for mammalian cell cytotoxicity than for antimicrobial activity (Table 1). The most active derivatives, **6g** and **6h**, displayed only modest selectivity towards *S. aureus* with selectivity index (SI) values of 3.5 and 2.9, respectively (Table 1).

The derivatives that were non-cytotoxic to mammalian cells at the highest tested concentration of 100 µM had only moderate antibacterial activity (MIC_90_ ≥ 75 µM and MIC_50_ ≥ 11.6 µM). The 4-phenyl-2-aminoimidazole derivative **7** exhibited an eight-fold selectivity between *S. aureus* and the mammalian cell line, but its antibacterial effect (MIC_90_ = 100 µM) was modest. The selectivity index of the 5-chloro-indole derivative **5i** was >8.6, due to its non-cytotoxicity against mammalian cells at 100 µM (the highest tested concentration). An SI value greater than 10 can be regarded as a threshold for considering a compounds’ potential for further development [31]; thus, improving the selectivity of the most promising analogues found in this study through structural optimization would be justified.

## 3. Experimental Section

### 3.1. Determination of Antimicrobial Activity

#### 3.1.1. Microbial Strains

Clinical control strains of *Enterococcus faecalis* (Gram-positive, ATCC 29212), *Staphylococcus aureus* (Gram-positive, ATCC 25923), *Escherichia coli* (Gram-negative, ATCC 25922) and a fungal strain, *Candida albicans* (ATCC 90028), were obtained from Microbiologics Inc. (St. Cloud, MN, USA) and used for the antimicrobial screening. Bacterial strains were grown on Mueller Hinton II agar (MHA, Becton Dickinson, Franklin Lakes, NJ, USA) slants and Mueller Hinton II broth (MHB, Becton Dickinson, Franklin Lakes, NJ, USA) and the *Candida* strain on Sabouraud dextrose agar (SDA, Becton Dickinson, Franklin Lakes, NJ, USA) plates. Media were prepared in MilliQ water, according to the manufacturer’s instructions, and autoclaved at 121 °C for 15 min. Prior to the assay, bacterial suspensions were prepared in MHB from fresh slant cultures and incubated at 37 °C for 16–20 h at 100 rpm. The *Candida* strain was grown on SDA plates at 28 °C for 18–24 h and suspended into sterile 0.9% saline for the assay.

#### 3.1.2. Microdilution Assay

Antimicrobial assays were performed by the broth microdilution method following the guidelines of the Clinical and Laboratory Standards Institute (CLSI) and European Committee on Antimicrobial Susceptibility Testing (EUCAST). Bacterial suspensions were prepared as described above and diluted with MHB to obtain a final inoculum of 5 × 10^5^ colony-forming units (CFU)/milliliters in the assay (determined on the basis of absorbance values at 620 nm previously calibrated against plate counts). The *Candida* suspension was prepared in sterile 0.9% saline solution, and the suspension was diluted in RPMI-1640 media (with l-glutamine, without NaHCO_3_ and supplemented with 2% glucose and 0.165 M MOPS, buffered to pH 7; Lonza, Basel, Switzerland) to yield a final inoculum of 2.5 × 10^3^ CFU/mL in the assay. Assays were carried out in clear 96-well microtiter plates and initiated by dispensing an equal volume of microbial suspension and sample solution diluted into the assay medium. The plates were incubated for 24 h at 37 °C (for *Candida*, the incubation was at 28 °C for 48 h) with agitation. Absorbance was measured at 620 nm with a plate reader at 0, 4 and 24 h with the bacteria and at 0, 24 and 48 h with *Candida*. The antimicrobial activity of the samples was calculated from the absorbance values by comparing to untreated controls and expressed as the percentage inhibition of growth. Reference antibiotics were used as positive controls on every assay plate (see Figure 2 for details). Compounds were initially assayed at a final concentration of 50 µM (*n* = 3), and those that showed >80% inhibition in the primary screen were tested further at several concentrations to confirm the activity and to determine the MIC_90_ and MIC_50_ values. The MIC_90_ was defined as the lowest concentrations that showed >90% inhibition of growth. MIC_50_ values were determined from the dose-response results by sigmoidal curve fitting with Origin software (OriginLab, Corp., Wellesley Hills, MA, USA).

### 3.2. Determination of Mammalian Cell Cytotoxicity

#### 3.2.1. Cell Culture

Huh-7 cells (originating from human hepatocellular carcinoma) were kindly provided by Prof. Ralf Bartenschlager (University of Heidelberg, Heidelberg, Germany). The cells were cultured in Dulbecco’s Modified Eagle Medium (DMEM) supplemented with 10% fetal bovine serum (FBS, Gibco, Grand Island, NY, USA), 100 µM non-essential amino acids, 2 mM l-glutamine and 100 µg/mL of streptomycin and 100 IU/mL of penicillin (Gibco). The cells were incubated at 37 °C in a humidified atmosphere with 5% CO_2_.

#### 3.2.2. ATP Assay

The effect of the most promising compounds on the metabolic activity of Huh-7 hepatocytes was assessed by intracellular ATP quantitation (Promega’s CellTiter-Glo Cell Viability Assay, Madison, WI, USA). In brief, cells were seeded at 20,000 cells/well on white-walled 96-well microplates (ViewPlate, PerkinElmer Inc., Wellesley, MA, USA) and incubated at 37 °C, 5% CO_2_ and 95% humidity overnight and then exposed to the compounds for 24 h. Following the exposure, cells were washed with 100 µL PBS, and 50 µL of fresh assay media and 50 µL of the CellTiter-Glo reagent were added into the wells. After 2 min of shaking and 10 min incubation at rt, luminometric signal was measured using a Varioskan Flash plate reader (Thermo Fisher Scientific, Vantaa, Finland). Polymyxin-B sulfate (15,000 IU/mL, average cytotoxicity 88%) was used as a positive control on every assay plate. Compounds were primarily screened at 100 and 50 µM (*n* = 3) concentrations, and those that showed >50% cytotoxicity were further subjected to dose-response experiments to determine IC_50_ values (concentration ranging between 150 µM and 1.56 µM, depending on the potency of the compound). The IC_50_ values were calculated by fitting the data into sigmoidal dose-response curves with the Origin software.

### 3.3. Chemistry: General

Chemicals were obtained from Acros Organics (Geel, Belgium) and Sigma-Aldrich Corporation (St. Louis, MO, USA) and used without further purification. Analytical TLC was performed on silica gel Merck 60 F_254_ plates (0.25 mm), using visualization with UV light and ninhydrin. Column chromatography was carried out on silica gel 60 (particle size 240–400 mesh). HPLC analyses were performed on an Agilent Technologies 1100 instrument (Agilent Technologies, Santa Clara, CA, USA) with a G1365B UV-Vis detector, a G1316A thermostat and a G1313A autosampler using a Phenomenex Luna 5-μm C18 column (4.6 × 150 mm or 4.6 × 250 mm) (Phenomenex, Torrance, CA, USA) and a flow rate of 1.0 mL/min. The eluent consisted of trifluoroacetic acid (0.1% in water) or ammonia (0.1% in water) as solvent A and methanol as solvent B. Microwave-assisted reactions were performed using a CEM Discover microwave reactor (CEM Corp., Matthews, NC, USA). Melting points were determined on a Reichert hot stage microscope and are uncorrected. ^1^H, ^13^C and ^19^F NMR spectra were recorded at 400, 100 and 376 MHz, respectively, on a Bruker AVANCE III 400 spectrometer (Bruker Corporation, Billerica, MA, USA) in DMSO-*d*_6_, MeOH-*d*_4_ or acetone-*d*_6_ solutions, with TMS as the internal standard. IR spectra were recorded on a PerkinElmer Spectrum BX FT-IR spectrometer (PerkionElmer, Inc., Waltham, MA, USA) or Thermo Nicolet Nexus 470 ESP FT-IR spectrometer (Thermo Fisher Scientific, Waltham, MA, USA). Mass spectra were obtained using a VG Analytical Autospec Q mass spectrometer (Fisons, VG Analytical, Manchester, UK). The purity of the tested compounds was established to be ≥95%.

### 3.4. Synthetic Procedures

#### 3.4.1. General Procedure A: Synthesis of compounds **2a**–**d**

The corresponding carboxylic acid (0.36 mmol), TBTU (137 mg, 0.414 mmol) and *N*-methylmorpholine (0.08 mL, 0.72 mmol) were dissolved in dry dimethylformamide (2 mL) and stirred under argon at rt for 1 h. The prepared mixture was added dropwise to a stirred solution of compound **1** (50 mg, 0.36 mmol) and *N*-methylmorpholine (0.08 mL, 0.72 mmol) in dry dimethylformamide (1 mL) at 0 °C. After 1 h, the mixture was warmed to rt and stirred under argon for 5 h. The solvent was evaporated under reduced pressure, and the residue was purified by flash column chromatography using dichloromethane/methanol saturated with NH_3_ (6:1) as an eluent.

(*E*)-*N*-(3-(2-amino-1*H*-imidazol-4-yl)allyl)-1*H*-pyrrole-2-carboxamide (**2a**) (see Supplementary Information, Figure S1). Yield, 18%; brown solid; mp 95–98 °C; IR (KBr) ν = 3208 (N-H), 2927 (C-H), 1614 (C=O), 1558, 1520, 1406, 1323, 1197, 1113, 1040, 959, 884, 740 cm^−1^. ^1^H NMR (MeOH-*d*_4_) δ 4.05 (dd, 2H, *J* = 6.0 Hz, *J* = 1.2 Hz, -CH=CH-CH_2_-), 5.94 (dt,1H, *J* = 15.8 Hz, *J* = 6.0 Hz, -CH=CH-CH_2_-), 6.18 (dd, 1H, *J* = 3.7 Hz, *J* = 2.6 Hz, Ar-H^4^), 6.32 (td, 1H, *J* = 15.8 Hz, *J* = 1.2 Hz, -CH=CH-CH_2_-), 6.51 (s, 1H, imidazole-H), 6.82 (dd, 1H, *J* = 3.7 Hz, *J* = 1.4 Hz, Ar-H^3^), 6.93 (dd, 1H, *J* = 2.5 Hz, *J* = 1.4 Hz, Ar-H^5^); ^13^C NMR (MeOH-*d*_4_) δ 42.06, 110.22, 111.78, 117.01, 121.87, 122.66, 122.89, 126.87, 130.82, 151.66, 163.61; HRMS for C_11_H_13_N_5_O: calculated, 231.1120; found, 231.1189. HPLC: Phenomenex Luna 5 μm C18 column (4.6 mm × 150 mm); mobile phase: 10%–70% of MeOH in NH_3(aq)_ (0.1%) in 20 min; flow rate: 1.0 mL/min; injection volume: 10 μL; retention time: 10.11 min (97.9% at 254 nm, 98.2% at 280 nm).

(*E*)-*N*-(3-(2-amino-1*H*-imidazol-4-yl)allyl)-4,5-dibromo-1*H*-pyrrole-2-carboxamide (**2b**) (see Supplementary Information, Figure S2). Yield, 25%; yellow solid; mp 201–204 °C; IR (KBr) ν = 3117 (N-H), 2934 (C-H), 1611 (C=O), 1562, 1515, 1410, 1389, 1320, 1214, 1023, 955, 818, 754 cm^−1^. ^1^H NMR (MeOH-*d*_4_) δ 4.03 (d, 2H, *J* = 6.0 Hz, -CH=CH-CH_2_-), 5.91 (dt, 1H, *J* = 15.8 Hz, *J* = 6.0 Hz, -CH=CH-CH_2_-), 6.31 (d, 1H, *J* = 15.8 Hz, -CH=CH-CH_2_-), 6.51 (s, 1H, imidazole-H), 6.85 (s, 1H, Ar-H^3^); ^13^C NMR (MeOH-*d*_4_) δ 42.18, 99.96, 106.09, 114.29, 117.00, 122.12, 122.28, 128.88, 130.94, 151.72, 161.53; HRMS for C_11_H_11_Br_2_N_5_O: calculated, 386.9330; found, 386.9408. HPLC: Phenomenex Luna 5 μm C18 column (4.6 mm × 150 mm); mobile phase: 10%–70% of MeOH in NH_3(aq)_ (0.1%) in 20 min; flow rate: 1.0 mL/min; injection volume: 10 μL; retention time: 13.69 min (98.7% at 254 nm, 98.5% at 280 nm).

(*E*)-*N*-(3-(2-amino-1*H*-imidazol-4-yl)allyl)-1*H*-indole-2-carboxamide (**2c**) (see Supplementary Information, Figure S3). Yield, 26%; white solid; mp ˃ 230 °C; IR (KBr) ν = 3205 (N-H), 2930 (C-H), 1618 (C=O), 1546, 1418, 1340, 1308, 1258, 1100, 958, 812, 745 cm^−1^. ^1^H NMR (MeOH-*d*_4_) δ 4.12 (dd, 2H, *J* = 6.2 Hz, *J* = 1.3 Hz, -CH=CH-CH_2_-), 5.96 (ddd, 1H, *J* = 15.8 Hz, *J* = 6.2 Hz, *J* = 5.8 Hz, -CH=CH-CH_2_-), 6.36 (td, 1H, *J* = 15.8 Hz, *J* = 1.3 Hz, -CH=CH-CH_2_-), 6.50 (s, 1H, imidazole-H), 7.07 (ddd, 1H, *J* = 8.0 Hz, *J* = 7.0 Hz, *J* = 1.0, Ar-H^6^), 7.11 (d, 1H, *J* = 0.9 Hz, Ar-H^3^), 7.22 (ddd, 1H, *J* = 8.3 Hz, *J* = 7.0 Hz, *J* = 1.1, Ar-H^5^), 7.45 (ddd, 1H, *J* = 8.3 Hz, *J* = 1.8 Hz, *J* = 0.9, Ar-H^4^), 7.61 (td, 1H, *J* = 8.1 Hz, *J* = 1.0 Hz, Ar-H^7^); ^13^C NMR (MeOH-*d*_4_) δ 41.01, 102.99, 111.64, 116.17, 119.74, 120.24, 121.27, 121.34, 123.61, 127.62, 129.83, 130.82, 136.90, 150.54, 162.56; HRMS for C_15_H_15_N_5_O: calculated, 281.1277; found, 281.1344. HPLC: Phenomenex Luna 5 μm C18 column (4.6 mm × 150 mm); mobile phase: 10%–70% of MeOH in NH_3(aq)_ (0.1%) in 20 min; flow rate: 1.0 mL/min; injection volume: 10 μL; retention time: 17.29 min (99.2% at 254 nm, 99.4% at 280 nm).

(*E*)-*N*-(3-(2-amino-1*H*-imidazol-4-yl)allyl)-5-fluoro-1*H*-indole-2-carboxamide (**2d**) (see Supplementary Information, Figure S4). Yield, 27%; yellow solid; mp 153–156 °C; IR (KBr) ν = 3227 (N-H), 2928 (C-H), 1618 (C=O), 1544, 1485, 1420, 1326, 1258, 1226, 1158, 1109, 954, 856, 798, 757, 730 cm^−1^. ^1^H NMR (MeOH-*d*_4_) δ 4.11 (dd, 2H, *J* = 6.2 Hz, *J* = 1.3 Hz, -CH=CH-CH_2_-), 5.95 (td, 1H, *J* = 15.8 Hz, *J* = 6.2 Hz, -CH=CH-CH_2_-), 6.36 (td, 1H, *J* = 15.8 Hz, *J* = 1.3 Hz, -CH=CH-CH_2_-), 6.50 (s, 1H, imidazole-H), 7.02 (ddd, 1H, *J* = 8.0 Hz, *J* = 6.9 Hz, *J* = 0.9, Ar-H^6^), 7.08 (d, 1H, *J* = 0.9 Hz, Ar-H^3^), 7.28 (ddd, 1H, *J* = 9.6 Hz, *J* = 2.1 Hz, *J* = 0.4, Ar-H^4^), 7.43 (tdd, 1H, *J* = 9.0 Hz, *J* = 4.5 Hz, *J* = 0.7 Hz, Ar-H^7^); ^13^C NMR (MeOH-*d*_4_) δ 40.96, 102.82 (d, ^4^*J*_C-F_ = 5.2 Hz, C-4), 105.28 (d, ^2^*J*_C-F_ = 23.2 Hz, C-8), 112.26 (d, ^2^*J*_C-F_ = 27.0 Hz, C-2), 112.76 (d, ^3^*J*_C-F_ = 9.6 Hz, C-7), 115.93, 120.71, 120.96, 127.73 (d, ^3^*J*_C-F_ = 10.3 Hz, C-3), 129.32, 132.58, 133.51, 150.19, 157.96 (d, ^1^*J*_C-F_ =234.0 Hz, C-1), 162.19; HRMS for C_15_H_14_FN_5_O: calculated, 299.1182; found, 299.1194. HPLC: Phenomenex Luna 5 μm C18 column (4.6 mm × 150 mm); mobile phase: 10%–70% of MeOH in NH_3(aq)_ (0.1%) in 20 min; flow rate: 1.0 mL/min; injection volume: 10 μL; retention time: 18.61 min (98.8% at 254 nm, 99.0% at 280 nm).

#### 3.4.2. General Procedure B: Synthesis of Compounds **5a**–**l** and **10a**–**c** (with **5f** as an Example)

To a suspension of 5-methoxy-indole-2-carboxylic acid (251 mg, 1.31 mmol) and TBTU (456 mg, 1.42 mmol) in dichloromethane (5 mL), *N*-methylmorpholine (0.601 mL, 5.47 mmol) was added and the mixture stirred at rt for 0.5 h upon which a clear solution formed. Compound **3 **(300 mg, 1.09 mmol) was added and the mixture stirred at 35 °C for 24 h. The solvent was evaporated *in vacuo*, the residue dissolved in ethyl acetate (30 mL) and washed successively with water (2 × 10 mL), saturated aqueous NaHCO_3_ solution (2 × 10 mL) and brine (1 × 10 mL). The organic phase was dried over Na_2_SO_4_, filtered and the solvent evaporated under reduced pressure. The crude product was purified by flash column chromatography using ethyl acetate/petroleum ether or dichloromethane/methanol as an eluent, to afford **5f** (305 mg, 62% yield) as a white solid. Analytical and spectroscopic data for compounds **5a**–**e**, **5l** and **10a**–**c** are reported elsewhere [29].

*tert*-Butyl 2-amino-4-(3-(5-methoxy-1*H*-indole-2-carboxamido)phenyl)-1*H*-imidazole-1-carboxylate (**5f**) (see Supplementary Information, Figure S5). Yield, 62%; white solid; mp 173–177 °C; IR (KBr) ν = 3415 (N-H), 3299 (N-H), 3115 (C-H), 2991 (C-H), 2831 (C-H), 1739 (C=O), 1639, 1597, 1536, 1453, 1433, 1354, 1323, 1275, 1238, 1218, 1153, 1117, 1032, 976, 897, 847, 791, 758, 718 cm^−1^. ^1^H NMR (DMSO-*d*_6_) δ 1.60 (s, 9H, *t*-Bu), 3.79 (s, 3H, OCH_3_), 6.64 (s, 2H, NH_2_), 6.89 (dd, 1H, ^3^*J* = 9.2 Hz, ^4^*J* = 2.4 Hz, Ar-H), 7.14 (d, 1H, ^4^*J* = 2.4 Hz, Ar-H), 7.29–7.38 (m, 4H, 4 × Ar-H), 7.48 (dd, 1H, ^3^*J* = 7.6 Hz, ^4^*J* = 0.8 Hz, Ar-H), 7.72–7.75 (m, 1H, Ar-H), 8.13 (s, 1H, Ar-H), 10.19 (s, 1H, NH), 11.57 (s, 1H, NH); ^13^C NMR (DMSO-*d*_6_) δ 27.51 (CCH_3_), 55.25 (OCH_3_), 84.70 (CCH_3_), 102.04, 103.52, 106.07, 113.19, 115.04, 116.55, 118.81, 119.92, 127.35, 128.69, 131.74, 132.08, 133.80, 136.95, 139.12, 148.88, 150.43, 153.82, 159.61; MS (ESI) *m*/*z* (%) = 448.2 (MH^+^, 15), 392.1 ([MH-*t*-Bu]H^+^, 90), 348.1 ([MH-Boc]H^+^, 100). HRMS for C_24_H_26_N_5_O_4_: calculated, 448.1985; found, 448.1983. HPLC: Phenomenex Luna 5 μm C18 column (4.6 mm × 150 mm); mobile phase: 10%–90% of MeOH in TFA (0.1%) in 20 min; flow rate: 1.0 mL/min; injection volume: 10 μL; retention time: 16.515 min (98.2% at 254 nm, 98.9% at 280 nm).

*tert*-Butyl 2-amino-4-(3-(5-(trifluoromethoxy)-1*H*-indole-2-carboxamido)phenyl)-1*H*-imidazole-1-carboxylate (**5g**) (see Supplementary Information, Figure S6). Yield, 76%; white solid; mp 185–187 °C; IR (KBr) ν = 3434 (N-H), 3368 (N-H), 3100 (C-H), 3006 (C-H), 1721 (C=O), 1649, 1600, 1548, 1435, 1364, 1257, 1233, 1217, 1206, 1152, 1119, 1064, 977, 895, 877, 854, 794, 775, 760, 715 cm^−1^. ^1^H NMR (DMSO-*d*_6_) δ 1.60 (s, 9H, *t*-Bu), 6.64 (s, 2H, NH_2_), 7.22 (dd, 1H, ^3^*J* = 9.2 Hz, ^4^*J* = 1.6 Hz, Ar-H), 7.30 (s, 1H, Ar-H), 7.35 (t, 1H, ^3^*J* = 8.0 Hz, Ar-H), 7.49–7.57 (m, 3H, 3 × Ar-H), 7.74–7.76 (m, 2H, 2 × Ar-H), 8.14 (s, 1H, Ar-H), 10.36 (s, 1H, NH), 12.01 (s, 1H, NH); ^13^C NMR (DMSO-*d*_6_) δ 27.51 (CCH_3_), 84.70 (CCH_3_), 104.09, 106.13, 113.61, 113.96, 116.63, 117.63, 118.88, 120.15, 120.41 (q, 1C, ^1^*J*_C-F_ = 253 Hz, CF_3_), 127.02, 128.73, 133.56, 133.85, 135.15, 136.89, 138.90, 142.20, 148.87, 150.43, 159.22; ^19^F NMR (DMSO-*d*_6_) δ −59.92 (s, 3F, CF_3_); MS (ESI) *m*/*z* (%) = 502.2 (MH^+^, 10), 446.1 ([MH-*t*-Bu]H^+^, 90), 402.1 ([MH-Boc]H^+^, 100). HRMS for C_24_H_23_N_5_O_4_F_3_: calculated, 502.1702; found, 502.1712. HPLC: Phenomenex Luna 5 μm C18 column (4.6 mm × 150 mm); mobile phase: 60%–90% of MeOH in TFA (0.1%) in 20 min; flow rate: 1.0 mL/min; injection volume: 10 μL; retention time: 6.886 min (99.5% at 254 nm, 99.2% at 280 nm).

*tert*-Butyl 2-amino-4-(3-(5-(benzyloxy)-1*H*-indole-2-carboxamido)phenyl)-1*H*-imidazole-1-carboxylate (**5h**) (see Supplementary Information, Figure S7). Yield, 42%; an off-white solid; mp 140–143 °C; IR (KBr) ν = 3439 (N-H), 3385 (N-H), 3116 (C-H), 3064 (C-H), 2984 (C-H), 1724 (C=O), 1642, 1624, 1596, 1539, 1433, 1361, 1274, 1246, 1232, 1210, 1156, 1116, 1067, 1015, 977, 841, 800, 758, 745, 732, 698 cm^−1^. ^1^H NMR (DMSO-*d*_6_) δ 1.60 (s, 9H, *t*-Bu), 5.13 (s, 2H, OCH_2_), 6.64 (s, 2H, NH_2_), 6.97 (dd, 1H, ^3^*J* = 8.8 Hz, ^4^*J* = 2.4 Hz, Ar-H), 7.25–7.51 (m, 11H, 11 × Ar-H), 7.72–7.75 (m, 1H, Ar-H), 8.14 (s, 1H, Ar-H), 10.18 (s, 1H, NH), 11.59 (s, 1H, NH); ^13^C NMR (DMSO-*d*_6_) δ 27.51 (CCH_3_), 69.61 (OCH_2_), 84.70 (CCH_3_), 103.54, 103.67, 106.07, 113.21, 115.53, 116.51, 118.77, 119.92, 127.30, 127.68, 128.36, 128.69, 131.84, 132.22, 133.80, 136.95, 137.54, 139.12, 148.88, 150.43, 152.81, 159.59 (signals for two C atoms overlap); MS (ESI) *m*/*z* (%) = 524.2 (MH^+^, 40), 468.2 ([MH-*t*-Bu]H^+^, 80), 424.2 ([MH-Boc]H^+^, 100). HRMS for C_30_H_30_N_5_O_4_: calculated, 524.2298; found, 524.2302. HPLC: Phenomenex Luna 5 μm C18 column (4.6 mm × 150 mm); mobile phase: 60%–90% of MeOH in TFA (0.1%) in 20 min; flow rate: 1.0 mL/min; injection volume: 10 μL; retention time: 7.723 min (96.8% at 254 nm, 98.4% at 280 nm).

*tert*-Butyl 2-amino-4-(3-(5-chloro-1*H*-indole-2-carboxamido)phenyl)-1*H*-imidazole-1-carboxylate (**5i**) (see Supplementary Information, Figure S8). Yield, 65%; white solid; mp 188–190 °C; IR (KBr) ν = 3,414 (N-H), 3,371 (N-H), 3,146 (C-H), 2,981 (C-H), 1,725 (C=O), 1,648, 1,600, 1,544, 1,434, 1,372, 1,360, 1,317, 1,283, 1,243, 1,223, 1,204, 1,157, 1,117, 1,059, 977, 915, 874, 855, 793, 759, 712 cm^−1^. ^1^H NMR (DMSO-*d*_6_) δ 1.60 (s, 9H, *t*-Bu), 6.64 (s, 2H, NH_2_), 7.24 (dd, 1H, ^3^*J* = 8.8 Hz, ^4^*J* = 2.0 Hz, Ar-H), 7.30 (s, 1H, Ar-H), 7.35 (t, 1H, ^3^*J* = 8.0 Hz, Ar-H), 7.45–7.50 (m, 3H, 3 × Ar-H), 7.72–7.75 (m, 1H, Ar-H), 7.79 (d, 1H, ^4^*J* = 2.0 Hz, Ar-H), 8.14 (t, 1H, ^4^*J* = 1.6 Hz, Ar-H), 10.32 (s, 1H, NH), 11.94 (s, 1H, NH); ^13^C NMR (DMSO-*d*_6_) δ 27.51 (CCH_3_), 84.70 (CCH_3_), 103.34, 106.11, 113.96, 116.58, 118.83, 120.11, 120.80, 123.85, 124.36, 128.07, 128.73, 132.97, 133.84, 135.14, 136.90, 138.95, 148.87, 150.44, 159.28; MS (ESI) *m*/*z* (%) = 452.1 (MH^+^, 15), 396.1 ([MH-*t*-Bu]H^+^, 100), 352.1 ([MH-Boc]H^+^, 40). HRMS for C_23_H_23_N_5_O_3_Cl: calculated, 452.1489; found, 452.1487. HPLC: Phenomenex Luna 5 μm C18 column (4.6 mm × 150 mm); mobile phase: 60%–90% of MeOH in TFA (0.1%) in 20 min; flow rate: 1.0 mL/min; injection volume: 10 μL; retention time: 5.299 min (98.3% at 254 nm, 98.6% at 280 nm).

*tert*-Butyl 2-amino-4-(3-(5-fluoro-1*H*-indole-2-carboxamido)phenyl)-1*H*-imidazole-1-carboxylate (**5j**) (see Supplementary Information, Figure S9). Yield, 76%; white solid; mp 189–192 °C; IR (KBr) ν = 3450 (N-H), 3409 (N-H), 3280 (N-H), 3113 (C-H), 2976 (C-H), 1726 (C=O), 1651, 1610, 1597, 1545, 1489, 1446, 1426, 1356, 1330, 1314, 1259, 1210, 1159, 1146, 1117, 1067, 955, 850, 791, 718 cm^−1^. ^1^H NMR (DMSO-*d*_6_) δ 1.60 (s, 9H, *t*-Bu), 6.64 (s, 2H, NH_2_), 7.10 (dt, 1H, ^3^*J* = 9.2 Hz, ^4^*J* = 2.4 Hz, Ar-H), 7.30 (s, 1H, Ar-H), 7.34 (t, 1H, ^3^*J* = 8.0 Hz, Ar-H), 7.45–7.50 (m, 4H, 4 × Ar-H), 7.72–7.75 (m, 1H, Ar-H), 8.14 (s, 1H, Ar-H), 10.29 (s, 1H, NH), 11.84 (s, 1H, NH); ^13^C NMR (DMSO-*d*_6_) δ 27.51 (CCH_3_), 84.70 (CCH_3_), 103.79 (d, 1C, ^4^*J*_C-F_ = 5 Hz), 105.88 (d, 1C, ^2^*J*_C-F_ = 23 Hz), 106.11, 112.51 (d, 1C, ^2^*J*_C-F_ = 27 Hz), 113.57 (d, 1C, ^3^*J*_C-F_ = 9 Hz), 116.58, 118.83, 120.08, 127.09 (d, 1C, ^3^*J*_C-F_ = 10 Hz), 128.72, 133.17, 133.50, 133.84, 136.91, 138.98, 148.87, 150.43, 157.19 (d, 1C, ^1^*J*_C-F_ = 231 Hz), 159.35; ^19^F NMR (DMSO-*d*_6_) δ −123.68 (s, 1F); MS (ESI) *m*/*z* (%) = 436.2 (MH^+^, 100). HRMS for C_23_H_23_N_5_O_3_F: calculated, 436.1785; found, 436.1780. HPLC: Phenomenex Luna 5 μm C18 column (4.6 mm × 150 mm); mobile phase: 10%–90% of MeOH in TFA (0.1%) in 20 min; flow rate: 1.0 mL/min; injection volume: 10 μL; retention time: 17.233 min (99.2% at 254 nm, 97.3% at 280 nm).

*tert*-Butyl 4-(3-(4*H*-thieno[3,2-*b*]pyrrole-5-carboxamido)phenyl)-2-amino-1*H*-imidazole-1-carboxylate (**5k**) (see Supplementary Information, Figure S10). Yield, 34%; off-white solid; mp 178–180 °C; IR (KBr) ν = 3,402 (N-H), 3,365 (N-H), 3,269 (N-H), 3154 (C-H), 2979 (C-H), 2933 (C-H), 1740 (C=O), 1635, 1596, 1541, 1519, 1460, 1428, 1352, 1311, 1256, 1239, 1207, 1153, 1118, 977, 895, 843, 827, 756, 717 cm^−1^. ^1^H NMR (acetone-*d*_6_) δ 1.67 (s, 9H, *t*-Bu), 6.44 (s, 2H, NH_2_), 7.09 (d, 1H, *J* = 5.2 Hz, Ar-H), 7.29–7.33 (m, 2H, 2 × Ar-H), 7.40–7.43 (m, 2H, 2 × Ar-H), 7.48–7.51 (m, 1H, Ar-H), 7.76–7.79 (m, 1H, Ar-H), 8.16 (s, 1H, Ar-H), 9.44 (s, 1H, NH), 11.07 (s, 1H, NH); ^13^C NMR (acetone-*d*_6_) δ 28.10 (CCH_3_), 85.61 (CCH_3_), 103.42, 107.05, 112.65, 117.25, 119.36, 120.84, 124.93, 128.62, 129.52, 132.06, 135.32, 138.56, 140.40, 142.30, 150.40, 151.58, 160.34; MS (ESI) *m*/*z* (%) = 424.2 (MH^+^, 10), 368.1 ([MH-*t*-Bu]H^+^, 100), 324.1 ([MH-Boc]H^+^, 50). HRMS for C_21_H_22_N_5_O_3_S: calculated, 424.1443; found, 424.1450. HPLC: Phenomenex Luna 5 μm C18 column (4.6 mm × 150 mm); mobile phase: 10%–90% of MeOH in TFA (0.1%) in 20 min; flow rate: 1.0 mL/min; injection volume: 10 μL; retention time: 16.043 min (95.3% at 254 nm, 95.8% at 280 nm).

#### 3.4.3. *tert*-Butyl 2-Amino-4-(3-(5-hydroxy-1*H*-indole-2-carboxamido)phenyl)-1*H*-imidazole-1-carboxylate (7)

Compound **5h** (496 mg, 0.947 mmol) was dissolved in a mixture of THF (10 mL) and MeOH (15 mL), Pd/C (100 mg) was added and the reaction mixture stirred under hydrogen atmosphere for 10 h. The catalyst was filtered off, the solvent removed under reduced pressure and the crude product purified by flash column chromatography using ethyl acetate/petroleum ether as an eluent, to afford **7 **(see Supplementary Information, Figure S17) (275 mg, 67% yield) as an off-white solid; mp 192–196 °C; IR (KBr) ν = 3399 (N-H, O-H), 3272 (N-H, O-H), 3157 (C-H), 2979 (C-H), 1736 (C=O), 1625, 1597, 1538, 1432, 1391, 1353, 1319, 1276, 1212, 1154, 1119, 1062, 851, 786, 757, 717 cm^−1^. ^1^H NMR (DMSO-*d*_6_) δ 1.60 (s, 9H, *t*-Bu), 6.64 (br s, 2H, NH_2_), 6.78 (dd, 1H, ^3^*J* = 8.8 Hz, ^4^*J* = 2.4 Hz, Ar-H), 6.93 (d, 1H, ^4^*J* = 2.4 Hz, Ar-H), 7.26–7.35 (m, 4H, 4 × Ar-H), 7.47 (dd, 1H, ^3^*J* = 8.0 Hz, ^4^*J* = 1.6 Hz, Ar-H), 7.71–7.73 (m, 1H, Ar-H), 8.13–8.14 (m, 1H, Ar-H), 8.86 (s, 1H, OH), 10.12 (s, 1H, NH), 11.43 (s, 1H, NH); ^13^C NMR (DMSO-*d*_6_) δ 27.51 (CCH_3_), 84.69 (CCH_3_), 102.93, 104.35, 106.04, 112.85, 115.04, 116.45, 118.71, 119.85, 127.73, 128.68, 131.59, 131.60, 133.79, 136.97, 139.18, 148.88, 150.42, 151.19, 159.69; MS (ESI) *m*/*z* (%) = 434.2 (MH^+^, 10), 378.1 ([MH-*t*-Bu]H^+^, 100), 334.1 ([MH-Boc]H^+^, 50). HRMS for C_23_H_24_N_5_O_4_: calculated, 434.1828; found, 434.1823. HPLC: henomenex Luna 5 μm C18 column (4.6 mm × 150 mm); mobile phase: 10%–90% of MeOH in TFA (0.1%) in 20 min; flow rate: 1.0 mL/min; injection volume: 10 μL; retention time: 14.015 min (96.3% at 254 nm, 96.2% at 280 nm).

#### 3.4.4. General Procedure C: Synthesis of Compounds **6a**–**l**, **8** and **11a**–**c** (with **6f** as an Example)

A solution of compound **5f** (160 mg, 0.358 mmol) in a mixture of THF and EtOH = 1:2 (15 mL) was saturated with gaseous HCl and stirred at rt for 5 h. The solvent was removed under reduced pressure, the solid filtered off and washed with diethyl ether and dichloromethane, to afford **6f** (132 mg, 96% yield) as an off-white solid. Analytical and spectroscopic data for compounds **6a**–**e**, **6l** and **11a**–**c** are reported elsewhere [29].

2-Amino-4-(3-(5-methoxy-1*H*-indole-2-carboxamido)phenyl)-1*H*-imidazol-3-ium chloride (**6f**) (see Supplementary Information, Figure S11). Yield, 96%; off-white solid; mp 237–241 °C; IR (KBr) ν = 3301 (N-H), 3138 (C-H), 2955 (C-H), 2761 (C-H), 1673 (C=O), 1653, 1625, 1585, 1541, 1452, 1418, 1336, 1281, 1238, 1208, 1177, 1153, 1132, 1116, 1022, 883, 839, 788, 755 cm^−1^. ^1^H NMR (DMSO-*d*_6_) δ 3.79 (s, 3H, OCH_3_), 6.89 (dd, 1H, ^3^*J* = 9.2 Hz, ^4^*J* = 2.4 Hz, Ar-H), 7.15 (d, 1H, ^4^*J* = 2.4 Hz, Ar-H), 7.33 (s, 1H, Ar-H), 7.37–7.49 (m, 6H, 4 × Ar-H, NH_2_), 7.69–7.72 (m, 1H, Ar-H), 8.08 (s, 1H, Ar-H), 10.42 (s, 1H, NH), 11.70 (s, 1H, NH), 12.16 (s, 1H, NH), 12.85 (s, 1H, NH); ^13^C NMR (DMSO-*d*_6_) δ 55.25 (OCH_3_), 102.02, 104.14, 109.43, 113.23, 115.19, 116.46, 119.74, 120.33, 126.39, 127.26, 128.12, 129.30, 131.54, 132.16, 139.45, 147.82, 153.84, 159.72; MS (ESI) *m*/*z* (%) = 348.2 ([M − Cl]^+^, 100). HRMS for C_19_H_18_N_5_O_2_: calculated, 348.1461; found, 348.1459. HPLC: Phenomenex Luna 5 μm C18 column (4.6 mm × 150 mm); mobile phase: 60%–90% of MeOH in TFA (0.1%) in 20 min; flow rate: 1.0 mL/min; injection volume: 10 μL; retention time: 3.029 min (98.2% at 254 nm, 98.7% at 280 nm).

2-Amino-4-(3-(5-(trifluoromethoxy)-1*H*-indole-2-carboxamido)phenyl)-1*H*-imidazol-3-ium chloride (**6g**) (see Supplementary Information, Figure S12). Yield, 66%; off-white solid; mp 255–260 °C; IR (KBr) ν = 3291 (N-H), 3148 (C-H), 3061 (C-H), 1677 (C=O), 1660, 1605, 1545, 1494, 1449, 1406, 1333, 1319, 1251, 1242, 1214, 1199, 1178, 1158, 1128, 1108, 1096, 970, 897, 868, 800, 788, 733 cm^−1^. ^1^H NMR (DMSO-*d*_6_) δ 7.22–7.25 (m, 1H, Ar-H), 7.34 (s, 1H, Ar-H), 7.41–7.58 (m, 6H, 4 × Ar-H, NH_2_), 7.68–7.71 (m, 1H, Ar-H), 7.74 (s, 1H, Ar-H), 8.07 (t, 1H, ^4^*J* = 1.6 Hz, Ar-H), 10.55 (s, 1H, NH), 12.11 (s, 1H, NH), 12.13 (s, 1H, NH), 12.81 (s, 1H, NH); ^13^C NMR (DMSO-*d*_6_) δ 104.71, 109.50, 113.67, 113.95, 116.58, 117.75, 120.00, 120.40 (q, 1C, ^1^*J*_C-F_ = 253 Hz, CF_3_), 120.44, 126.34, 126.92, 128.18, 129.35, 133.36, 135.21, 139.21, 142.21, 147.83, 159.34; ^19^F NMR (DMSO-*d*_6_) δ −56.93 (s, 3F, CF_3_); MS (ESI) *m*/*z* (%) = 402.1 ([M − Cl]^+^, 100). HRMS for C_19_H_15_N_5_O_2_F_3_: calculated, 402.1178; found, 402.1171. HPLC: Phenomenex Luna 5 μm C18 column (4.6 mm × 150 mm); mobile phase: 10%–90% of MeOH in TFA (0.1%) in 20 min; flow rate: 1.0 mL/min; injection volume: 10 μL; retention time: 19.439 min (96.3% at 254 nm, 97.0% at 280 nm).

2-Amino-4-(3-(5-(benzyloxy)-1*H*-indole-2-carboxamido)phenyl)-1*H*-imidazol-3-ium chloride (**6h**) (see Supplementary Information, Figure S13). Yield, 51%; off-white solid; mp 175–178 °C; IR (KBr) ν = 3253 (N-H), 3145 (C-H), 3032 (C-H), 2773 (C-H), 1678 (C=O), 1624, 1608, 1537, 1487, 1447, 1426, 1384, 1331, 1287, 1229, 1203, 1160, 1118, 1023, 940, 786, 757, 732 cm^−1^. ^1^H NMR (DMSO-*d*_6_) δ 5.13 (s, 2H, OCH_2_), 6.98 (dd, 1H, ^3^*J* = 9.2 Hz, ^4^*J* = 2.4 Hz, Ar-H), 7.26 (d, 1H, ^4^*J* = 2.4 Hz, Ar-H), 7.32–7.51 (m, 12H, 10 × Ar-H, NH_2_), 7.67–7.69 (m, 1H, Ar-H), 8.08 (t, 1H, ^4^*J* = 2.0 Hz, Ar-H), 10.37 (s, 1H, NH), 11.69 (s, 1H, NH), 12.12 (s, 1H, NH), 12.80 (s, 1H, NH); ^13^C NMR (DMSO-*d*_6_) δ 69.61 (OCH_2_), 103.67, 103.92, 109.59, 113.27, 115.69, 116.50, 119.85, 120.33, 126.49, 127.23, 127.68, 128.15, 128.37, 129.34, 131.59, 132.31, 137.52, 139.38, 147.74, 152.85, 159.72 (signals for two C atoms overlap); MS (ESI) *m*/*z* (%) = 424.2 ([M − Cl]^+^, 100). HRMS for C_25_H_22_N_5_O_2_: calculated, 424.1774; found, 424.1771. HPLC: Phenomenex Luna 5 μm C18 column (4.6 mm × 150 mm); mobile phase: 60%–90% of MeOH in TFA (0.1%) in 20 min; flow rate: 1.0 mL/min; injection volume: 10 μL; retention time: 7.803 min (95.3% at 254 nm, 95.1% at 280 nm).

2-Amino-4-(3-(5-chloro-1*H*-indole-2-carboxamido)phenyl)-1*H*-imidazol-3-ium chloride (**6i**) (see Supplementary Information, Figure S14). Yield, 71%; white solid; mp 201–204 °C; IR (KBr) ν = 3410 (N-H), 3260 (N-H), 3145 (C-H), 3032 (C-H), 2761 (C-H), 1693 (C=O), 1667, 1610, 1542, 1485, 1442, 1412, 1326, 1301, 1275, 1245, 1224, 1190, 1124, 1056, 914, 854, 798, 782, 754, 725 cm^−1^. ^1^H NMR (DMSO-*d*_6_) δ 7.25 (dd, 1H, ^3^*J* = 8.8 Hz, ^4^*J* = 2.0 Hz, Ar-H), 7.33 (s, 1H, Ar-H), 7.40–7.51 (m, 6H, 4 × Ar-H, NH_2_), 7.69–7.71 (m, 1H, Ar-H), 7.79 (d, 1H, ^4^*J* = 2.0 Hz, Ar-H), 8.07 (s, 1H, Ar-H), 10.53 (s, 1H, NH), 12.05 (s, 1H, NH), 12.14 (s, 1H, NH), 12.84 (s, 1H, NH); ^13^C NMR (DMSO-*d*_6_) δ 103.87, 109.52, 114.01, 116.53, 119.98, 120.40, 120.82, 123.98, 124.42, 126.37, 127.98, 128.18, 129.36, 132.75, 135.21, 139.23, 147.80, 159.41; MS (ESI) *m*/*z* (%) = 352.1 ([M − Cl]^+^, 100). HRMS for C_18_H_15_N_5_OCl: calculated, 352.0965; found, 352.0959. HPLC: Phenomenex Luna 5 μm C18 column (4.6 mm × 150 mm); mobile phase: 60%–90% of MeOH in TFA (0.1%) in 20 min; flow rate: 1.0 mL/min; injection volume: 10 μL; retention time: 5.338 min (98.4% at 254 nm, 98.8% at 280 nm).

2-Amino-4-(3-(5-fluoro-1*H*-indole-2-carboxamido)phenyl)-1*H*-imidazol-3-ium chloride (**6j**) (see Supplementary Information, Figure S15). Yield, 77%; off-white solid; mp 202–205 °C; IR (KBr) ν = 3443 (N-H), 3275 (N-H), 3145 (C-H), 2764 (C-H), 1662 (C=O), 1628, 1607, 1544, 1486, 1449, 1411, 1327, 1287, 1244, 1231, 1204, 1145, 1103, 954, 840, 780, 752, 727 cm^−1^. ^1^H NMR (DMSO-*d*_6_) δ 7.11 (dt, 1H, ^3^*J* = 9.2 Hz, ^4^*J* = 2.0 Hz, Ar-H), 7.33 (s, 1H, Ar-H), 7.40–7.50 (m, 7H, 5 × Ar-H, NH_2_), 7.69–7.71 (m, 1H, Ar-H), 8.08 (t, 1H, ^4^*J* = 1.6 Hz, Ar-H), 10.49 (s, 1H, NH), 11.95 (s, 1H, NH), 12.14 (s, 1H, NH), 12.83 (s, 1H, NH); ^13^C NMR (DMSO-*d*_6_) δ 104.34 (d, 1C, ^4^*J*_C-F_ = 5 Hz), 105.89 (d, 1C, ^2^*J*_C-F_ = 23 Hz), 109.51, 112.65 (d, 1C, ^2^*J*_C-F_ = 26 Hz), 113.63 (d, 1C, ^3^*J*_C-F_ = 9 Hz), 116.52, 119.93, 120.39, 126.37, 127.00 (d, 1C, ^3^*J*_C-F_ = 9 Hz), 128.17, 129.35, 132.96, 133.57, 139.28, 147.81, 157.20 (d, 1C, ^1^*J*_C-F_ = 231 Hz), 159.47; ^19^F NMR (DMSO-*d*_6_) δ −123.59 (s, 1F); MS (ESI) *m*/*z* (%) = 336.1 ([M − Cl]^+^, 100). HRMS for C_18_H_15_N_5_OF: calculated, 336.1261; found, 336.1264. HPLC: Phenomenex Luna 5 μm C18 column (4.6 mm × 150 mm); mobile phase: 60%–90% of MeOH in TFA (0.1%) in 20 min; flow rate: 1.0 mL/min; injection volume: 10 μL; retention time: 3.585 min (99.4% at 254 nm, 99.1% at 280 nm).

4-(3-(4*H*-Thieno[3,2-*b*]pyrrole-5-carboxamido)phenyl)-2-amino-1*H*-imidazol-3-ium chloride (**6k**) (see Supplementary Information, Figure S16). Yield, 78%; off-white solid; mp 198–202 °C; IR (KBr) ν = 3241 (N-H), 3135 (C-H), 3047 (C-H), 2763 (C-H), 1677 (C=O), 1625, 1541, 1488, 1460, 1385, 1348, 1308, 1231, 1191, 1115, 1084, 963, 877, 827, 748, 711 cm^−1^. ^1^H NMR (DMSO-*d*_6_) δ 7.03 (dd, 1H, ^3^*J* = 5.2 Hz, ^4^*J* = 0.8 Hz, Ar-H), 7.31 (s, 1H, Ar-H), 7.36–7.49 (m, 6H, 4 × Ar-H, NH_2_), 7.66–7.69 (m, 1H, Ar-H), 8.06 (t, 1H, ^4^*J* = 1.6 Hz, Ar-H), 10.24 (s, 1H, NH), 11.99 (s, 1H, NH), 12.14 (s, 1H, NH), 12.82 (s, 1H, NH); ^13^C NMR (DMSO-*d*_6_) δ 103.91, 109.43, 111.90, 116.27, 119.48, 120.15, 122.94, 126.45, 128.08, 128.28, 129.26, 130.49, 139.64, 141.32, 147.77, 159.41; MS (ESI) *m*/*z* (%) = 324.1 ([M − Cl]^+^, 100). HRMS for C_16_H_14_N_5_OS: calculated, 324.0919; found, 324.0911. HPLC: Phenomenex Luna 5 μm C18 column (4.6 mm × 150 mm); mobile phase: 60%–90% of MeOH in TFA (0.1%) in 20 min; flow rate: 1.0 mL/min; injection volume: 10 μL; retention time: 2.790 min (96.5% at 254 nm, 97.0% at 280 nm).

2-Amino-4-(3-(5-hydroxy-1*H*-indole-2-carboxamido)phenyl)-1*H*-imidazol-3-ium chloride (**8**) (see Supplementary Information, Figure S18). Yield, 85%; red solid; mp 248–252 °C; IR (KBr) ν = 3262 (N-H, O-H), 3151 (C-H), 3027 (C-H), 2758 (C-H), 1681 (C=O), 1651, 1632, 1586, 1540, 1446, 1419, 1340, 1316, 1281, 1233, 1204, 1152, 1123, 952, 850, 788, 753 cm^−1^. ^1^H NMR (DMSO-*d*_6_) δ 6.79 (dd, 1H, ^3^*J* = 8.8 Hz, ^4^*J* = 2.0 Hz, Ar-H), 6.94 (d, 1H, ^4^*J* = 2.0 Hz, Ar-H), 7.26–7.48 (m, 7H, 5 × Ar-H, NH_2_), 7.67–7.69 (m, 1H, Ar-H), 8.08 (s, 1H, Ar-H), 8.91 (s, 1H, OH), 10.32 (s, 1H, NH), 11.52 (s, 1H, NH), 12.12 (s, 1H, NH), 12.80 (s, 1H, NH); ^13^C NMR (DMSO-*d*_6_) δ 103.40, 104.34, 109.51, 112.89, 115.22, 116.39, 119.73, 120.24, 126.46, 127.65, 128.12, 129.31, 131.35, 131.66, 139.48, 147.77, 151.26, 159.82; MS (ESI) *m*/*z* (%) = 334.1 ([M − Cl]^+^, 100). HRMS for C_18_H_16_N_5_O_2_: calculated, 334.1304; found, 334.1296. HPLC: Phenomenex Luna 5 μm C18 column (4.6 mm × 150 mm); mobile phase: 60%–90% of MeOH in TFA (0.1%) in 20 min; flow rate: 1.0 mL/min; injection volume: 10 μL; retention time: 1.923 min (98.0% at 254 nm, 98.3% at 280 nm).

#### 3.4.5. 1-Benzyl-4-(3-nitrophenyl)-1*H*-imidazol-2-amine (**13**)

To the suspension of compound **12** (0.864 g, 4.00 mmol) in CH_3_CN (30 mL) were added K_2_CO_3_ (1.38 g, 6.00 mmol) and benzyl bromide (0.713 mL, 6.00 mmol). The mixture was stirred at 50 °C for 14 h. The solvent was removed under reduced pressure, the brown residue was suspended in ethyl acetate (80 mL) and washed with water (2 × 40 mL) and brine (1 × 40 mL). The organic phase was dried over Na_2_SO_4_, filtered and the solvent removed *in vacuo*. The crude product was purified by column chromatography with dichloromethane/methanol (20:1) as an eluent to afford **13** (see Supplementary Information, Figure S19) (539 mg, 44% yield) as a yellow solid; mp 189–193 °C; IR (KBr) ν = 3404 (N-H), 3126 (C-H), 1649, 1585, 1523, 1546, 1440, 1342, 1211, 1068, 886, 802, 759, 717 cm^−1^. ^1^H NMR (DMSO-*d*_6_) δ 5.02 (s, 2H, CH_2_), 5.84 (s, 2H, NH_2_), 7.26–7.32 (m, 3H, 3 × Ar-H), 7.35–7.39 (m, 2H, 2 × Ar-H), 7.40 (s, 1H, Ar-H), 7.55 (t, 1H, ^3^*J* = 8.0 Hz, Ar-H), 7.92–7.95 (m, 1H, Ar-H), 7.99–8.03 (m, 1H, Ar-H), 8.41–8.43 (m, 1H, Ar-H); ^13^C NMR (DMSO-*d*_6_) δ 47.30, 113.02, 117.51, 119.60, 127.37, 127.40, 128.54, 129.57, 129.74, 133.49, 137.04, 137.53, 148.23, 150.14; MS (ESI) *m*/*z* (%) = 295.1 (MH^+^, 100). HRMS for C_16_H_15_N_4_O_2_: calculated, 295.1195; found, 295.1199. HPLC: Phenomenex Luna 5 μm C18 column (4.6 mm × 150 mm); mobile phase: 10%–90% of MeOH in TFA (0.1%) in 20 min; flow rate: 1.0 mL/min; injection volume: 10 μL; retention time: 16.113 min (99.8% at 254 nm, 98.5% at 280 nm).

#### 3.4.6. 4-(3-Aminophenyl)-1-benzyl-1*H*-imidazol-2-amine (**14**)

Compound **13** (432 mg, 1.41 mmol) was dissolved in THF (50 mL), Pd/C (44 mg) was added and the reaction mixture was stirred under hydrogen atmosphere for 5 h. The catalyst was filtered off and the solvent removed under reduced pressure to yield **14** (see Supplementary Information, Figure S20) (379 mg, 97% yield) as an off-white solid; mp 192–195 °C; IR (KBr) ν = 3370 (N-H), 3067 (C-H), 1647, 1616, 1,569, 1545, 1481, 1438, 1363, 1338, 1271, 1202, 1129, 1067, 993, 976, 884, 819, 789, 732 cm^−1^. ^1^H NMR (DMSO-*d*_6_) δ 4.90 (s, 2H, NH_2_), 4.96 (s, 2H, CH_2_), 5.57 (s, 2H, NH_2_), 6.30–6.34 (m, 1H, Ar-H), 6.74–6.78 (m, 1H, Ar-H), 6.86 (t, 1H, ^4^*J* = 2.0 Hz, Ar-H), 6.89 (s, 1H, Ar-H), 6.90 (t, 1H, ^3^*J* = 7.8 Hz, Ar-H),7.23–7.30 (m, 3H, 3 × Ar-H), 7.33–7.38 (m, 2H, 2 × Ar-H); ^13^C NMR (DMSO-*d*_6_) δ 47.12, 109.56, 110.11, 111.41, 111.86, 127.28, 127.37, 128.47, 128.56, 135.66, 136.26, 137.89, 148.37, 149.25; MS (ESI) *m*/*z* (%) = 265.1 (MH^+^, 100). HRMS for C_16_H_17_N_4_: calculated, 265.1453; found, 265.1459. HPLC: Phenomenex Luna 5 μm C18 column (4.6 mm × 150 mm); mobile phase: 10%–90% of MeOH in TFA (0.1%) in 20 min; flow rate: 1.0 mL/min; injection volume: 10 μL; retention time: 11.334 min (95.3% at 254 nm, 96.6% at 280 nm).

#### 3.4.7. *N*-(3-(2-Amino-1-benzyl-1*H*-imidazol-4-yl)phenyl)-1*H*-pyrrole-2-carboxamide (**15**)

To a suspension of pyrrole-2-carboxylic acid (77 mg, 0.69 mmol) in dichloromethane (30 mL) were added triethylamine (0.192 mL, 1.38 mmol) and TBTU (244 mg, 0.76 mmol) and the mixture stirred at rt for 0.5 h upon which an opalescent solution formed. Compound **14 **(190 mg, 0.69 mmol) was added and the mixture stirred at rt for 18 h. The reaction mixture was diluted with dichloromethane (20 mL) and washed with saturated aqueous NaHCO_3_ solution (2 × 30 mL) and brine (1 × 40 mL). The organic phase was dried over Na_2_SO_4_, filtered and concentrated *in vacuo*. The crude product was purified by flash column chromatography using dichloromethane/methanol as an eluent, to afford **15** (see Supplementary Information, Figure S21) (105 mg, 41% yield) as an off-white solid; mp 229–232 °C; IR (KBr) ν = 3370 (N-H), 3066 (C-H), 1646 (C=O), 1606, 1570, 1545, 1496, 1454, 1437, 1364, 1308, 1202, 1169, 1130, 1068, 993, 896, 884, 823, 790, 725 cm^−1^. ^1^H NMR (DMSO-*d*_6_) δ 5.02 (s, 2H, CH_2_), 5.69 (s, 2H, NH_2_), 6.15–6.18 (m, 1H, Ar-H), 6.95–6.98 (m, 1H, Ar-H), 7.03 (s, 1H, Ar-H), 7.08–7.12 (m, 1H, Ar-H), 7.21 (t, 1H, ^3^*J* = 7.8 Hz, Ar-H), 7.26–7.32 (m, 4H, 4 × Ar-H), 7.35–7.40 (m, 2H, 2 × Ar-H), 7.54–7.58 (m, 1H, Ar-H), 7.94–7.97 (m, 1H, Ar-H), 9.70 (s, 1H, NH) 11.60 (br s, 1H, NH); ^13^C NMR (DMSO-*d*_6_) δ 47.20, 108.84, 110.73, 111.29, 115.37, 117.14, 118.45, 122.30, 126.18, 127.35, 127.44, 128.34, 128.52, 135.40, 135.49, 137.76, 139.34, 149.52, 158.99; MS (ESI) *m*/*z* (%) = 358.2 (MH^+^, 100). HRMS for C_21_H_20_N_5_O: calculated, 358.1668; found, 358.1661. HPLC: Phenomenex Luna 5 μm C18 column (4.6 mm × 150 mm); mobile phase: 10%–90% of MeOH in TFA (0.1%) in 20 min; flow rate: 1.0 mL/min; injection volume: 10 μL; retention time: 16.868 min (97.4% at 254 nm, 96.9% at 280 nm).

#### 3.4.8. 4-(3-(((1*H*-Pyrrol-2-yl)methyl)amino)phenyl)-1-benzyl-1*H*-imidazol-2-amine (**16**)

To a suspension of compound **14** (190 mg, 0.69 mmol) in dichloromethane (30 mL) were added pyrrole-2-carboxaldehyde (208 mg, 1.04 mmol) and glacial acetic acid (40 μL, 0.69 mmol) upon which the mixture became clear. NaBH(OAc)_3_ (208 mg, 1.04 mmol) was added and the mixture stirred at rt for 13 h. Red opalescent solution was diluted with dichloromethane (20 mL) and washed with saturated aqueous NaHCO_3_ solution (2 × 30 mL) and brine (1 × 30 mL). The organic phase was dried over Na_2_SO_4_, filtered and concentrated *in vacuo*. Crude product was recrystallized from ethyl acetate to give **16 **(see Supplementary Information, Figure S22)**¸**(120 mg, 49% yield) as red crystals; mp 165–168 °C; IR (KBr) ν = 3370 (N-H), 3066 (C-H), 1646, 1602, 1570, 1495, 1454, 1364, 1271, 1201, 1183, 1130, 1096, 993, 860, 790, 729 cm^−1^. ^1^H NMR (DMSO-*d*_6_) δ 4.14 (d, 2H, ^3^*J* = 5.5 Hz, CH_2_) 4.97 (s, 2H, CH_2_), 5.56–5.62 (m, 3H, NH, NH_2_), 5.91–5.96 (m, 2H, 2 × Ar-H), 6.40–6.44 (m, 1H, Ar-H), 6.62–6.65 (m, 1H, Ar-H), 6.80–6.84 (m, 1H, Ar-H), 6.93–6.98 (m, 3H, 3 × Ar-H), 7.23–7.31 (m, 3H, 3 × Ar-H), 7.33–7.39 (m, 2H, 2 × Ar-H), 10.70 (br s, 1H, NH); ^13^C NMR (DMSO-*d*_6_) δ 40.44, 47.12, 105.71, 107.11, 107.66, 110.20, 110.33, 112.09, 116.70, 127.27, 127.32, 128.47 (2 signals overlapped), 128.68, 135.62, 136.30, 137.90, 148.70, 149.29; MS (ESI) *m*/*z* (%) = 344.2 (MH^+^, 100). HRMS for C_21_H_22_N_5_: calculated, 344.1875; found, 344.1873. HPLC: Phenomenex Luna 5 μm C18 column (4.6 mm × 150 mm); mobile phase: 10%–90% of MeOH in TFA (0.1%) in 20 min; flow rate: 1.0 mL/min; injection volume: 10 μL; retention time: 11.474 min (97.9% at 254 nm, 97.4% at 280 nm).

## 4. Conclusions

In the present study, we have prepared the natural pyrrole-2-aminoimidazole alkaloids, clathrodin and oroidin, originally isolated from *Agelas* sponges and four families of their synthetic analogues. In total, 36 compounds were screened against a panel of laboratory strains, representing Gram-positive (*Enterococcus faecalis* and *Staphylococcus* *aureus*) and Gram-negative bacteria (*Escherichia coli*) and fungi (*Candida albicans*). Starting from the molecule of oroidin, with structural optimization using medicinal chemistry strategies, we have succeeded to prepare analogues with improved antimicrobial activities against all of the microbial strains tested. Twelve active compounds were selected, and their minimum inhibitory concentrations (MIC_50_, MIC_90_), as well as selectivities against mammalian cells were further determined. The most promising results were obtained for indole-based derivatives **6h** with MIC_90_ of 12.5 µM against the Gram-positive bacteria and 50 µM against *E. coli* and **6g** with MIC_90_ of 25 µM against all the bacteria and 50 µM against *C. albicans*. Although the effects were shown to be mostly broad-spectrum, targeting both prokaryotic and eukaryotic cells, in general, the IC_50_ values for mammalian cell cytotoxicity were slightly higher. Our results provide valuable information for future optimization towards a more selective antimicrobial compound.

## Abbreviations

ATCC: American Type Culture Collection
d: doublet
dd: doublet of doublets
ddd: doublet of doublet of doublets
DMF: *N*,*N*-dimethylformamide
DMSO: dimethylsulfoxide
dt: doublet of triplets
ESI: electrospray ionization
EtOH: ethanol
FT-IR: fourier transform infrared
HRMS: high resolution mass spectrometry
Huh-7: human hepatocellular carcinoma cell line
IR: infrared
MOPS: 3-(*N*-morpholino)propanesulfonic acid
mp: melting point
NMM: *N*-methylmorpholine
R: radical
RPMI-1640: Roswell Park Memorial Institute medium 1640
rt: room temperature
s: singlet
TBTU: *N,N,N′,N′*-tetramethyl-*O*-(benzotriazol-1-yl)uronium tetrafluoroborate
td: triplet of doublets
THF: tetrahydrofuran
TLC: thin layer chromatography
TMS: tetramethylsilane
Vis: visible light

## Supplementary Files

Supplementary File 1 Supplementary Information (PDF, 3708 KB)
