# Supplementary material for: Antimicrobial Activity of the Marine Alkaloids, Clathrodin and Oroidin, and Their Synthetic Analogues
Source: Mar Drugs. 2014 Feb 14;12(2):940–63. doi: 10.3390/md12020940 (PMC3944524; doi:10.3390/md12020940)

Supplementary Information

Figure S1. (*E*)-*N*-(3-(2-amino-1*H*-imidazol-4-yl)allyl)-1*H*-pyrrole-2-carboxamide (**2a**).
Figure S2. (*E*)-*N*-(3-(2-amino-1*H*-imidazol-4-yl)allyl)-4,5-dibromo-1*H*-pyrrole-2-carboxamide (**2b**).
Figure S3. (*E*)-*N*-(3-(2-amino-1*H*-imidazol-4-yl)allyl)-1*H*-indole-2-carboxamide (**2c**).
Figure S4. (*E*)-*N*-(3-(2-amino-1*H*-imidazol-4-yl)allyl)-5-fluoro-1*H*-indole-2-carboxamide (**2d**).
Figure S5. *tert*-Butyl 2-amino-4-(3-(5-methoxy-1*H*-indole-2-carboxamido)phenyl)-1*H*-imidazole-1-carboxylate (**5f**).
Figure S6. *tert*-Butyl 2-amino-4-(3-(5-(trifluoromethoxy)-1*H*-indole-2-carboxamido)phenyl)-1*H*-imidazole-1-carboxylate (**5g**).
Figure S7. *tert*-Butyl 2-amino-4-(3-(5-(benzyloxy)-1*H*-indole-2-carboxamido)phenyl)-1*H*-imidazole-1-carboxylate (**5h**).
Figure S8. *tert*-Butyl 2-amino-4-(3-(5-chloro-1*H*-indole-2-carboxamido)phenyl)-1*H*-imidazole-1-carboxylate (**5i**).
Figure S9. *tert*-Butyl 2-amino-4-(3-(5-fluoro-1*H*-indole-2-carboxamido)phenyl)-1*H*-imidazole-1-carboxylate (**5j**).
Figure S10. *tert*-Butyl 4-(3-(4*H*-thieno[3,2-*b*]pyrrole-5-carboxamido)phenyl)-2-amino-1*H*-imidazole-1-carboxylate (**5k**).
Figure S11. 2-Amino-4-(3-(5-methoxy-1*H*-indole-2-carboxamido)phenyl)-1*H*-imidazol-3-ium chloride (**6f**).
Figure S12. 2-Amino-4-(3-(5-(trifluoromethoxy)-1*H*-indole-2-carboxamido)phenyl)-1*H*-imidazol-3-ium chloride (**6g**).
Figure S13. 2-Amino-4-(3-(5-(benzyloxy)-1*H*-indole-2-carboxamido)phenyl)-1*H*-imidazol-3-ium chloride (**6h**).
Figure S14. 2-Amino-4-(3-(5-chloro-1*H*-indole-2-carboxamido)phenyl)-1*H*-imidazol-3-ium chloride (**6i**).
Figure S15. 2-Amino-4-(3-(5-fluoro-1*H*-indole-2-carboxamido)phenyl)-1*H*-imidazol-3-ium chloride (**6j**).
Figure S16. 4-(3-(4*H*-Thieno[3,2-*b*]pyrrole-5-carboxamido)phenyl)-2-amino-1*H*-imidazol-3-ium chloride (**6k**).
Figure S17. *tert*-Butyl 2-amino-4-(3-(5-hydroxy-1*H*-indole-2-carboxamido)phenyl)-1*H*-imidazole-1-carboxylate (**7**).
Figure S18. 2-Amino-4-(3-(5-hydroxy-1*H*-indole-2-carboxamido)phenyl)-1*H*-imidazol-3-ium chloride (**8**).
Figure S19. 1-Benzyl-4-(3-nitrophenyl)-1*H*-imidazol-2-amine (**13**).
Figure S20. 4-(3-Aminophenyl)-1-benzyl-1*H*-imidazol-2-amine (**14**).
Figure S21. *N*-(3-(2-Amino-1-benzyl-1*H*-imidazol-4-yl)phenyl)-1*H*-pyrrole-2-carboxamide (**15**).
Figure S22. 4-(3-(((1*H*-Pyrrol-2-yl)methyl)amino)phenyl)-1-benzyl-1*H*-imidazol-2-amine (**16**).

Figure S1. (*E*)-*N*-(3-(2-amino-1*H*-imidazol-4-yl)allyl)-1*H*-pyrrole-2-carboxamide (**2a**).

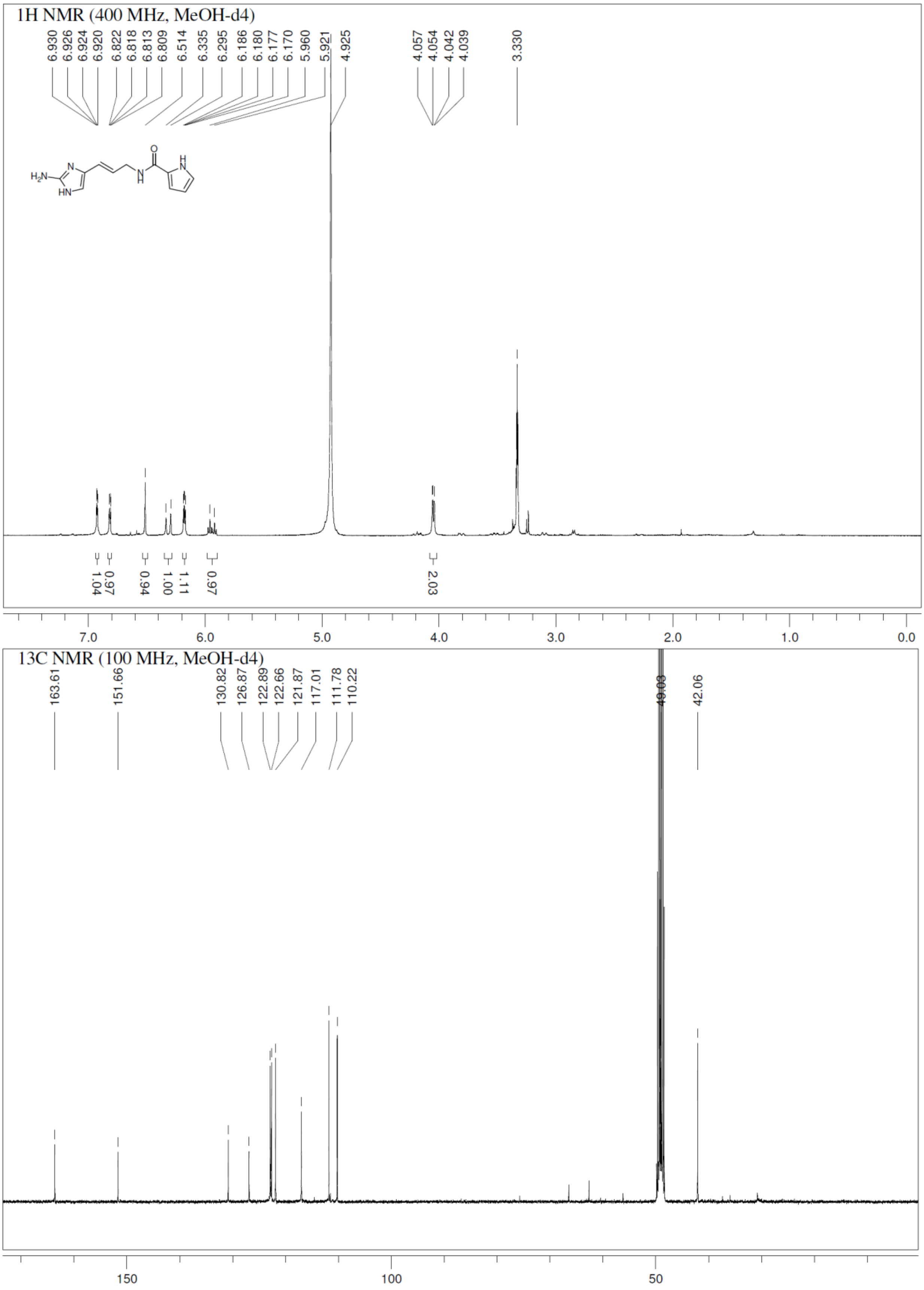

Figure S2. (*E*)-*N*-(3-(2-amino-1*H*-imidazol-4-yl)allyl)-4,5-dibromo-1*H*-pyrrole-2-carboxamide (**2b**).

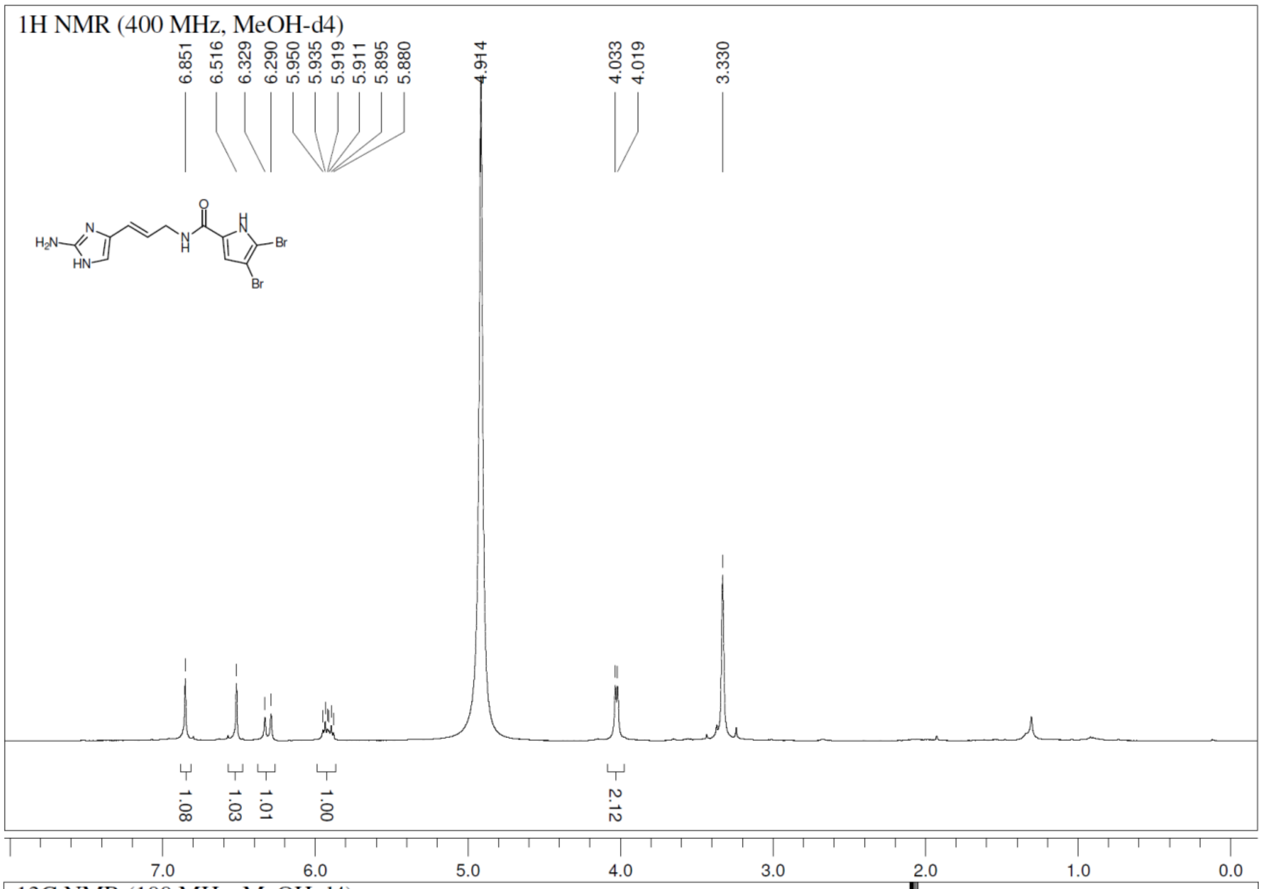

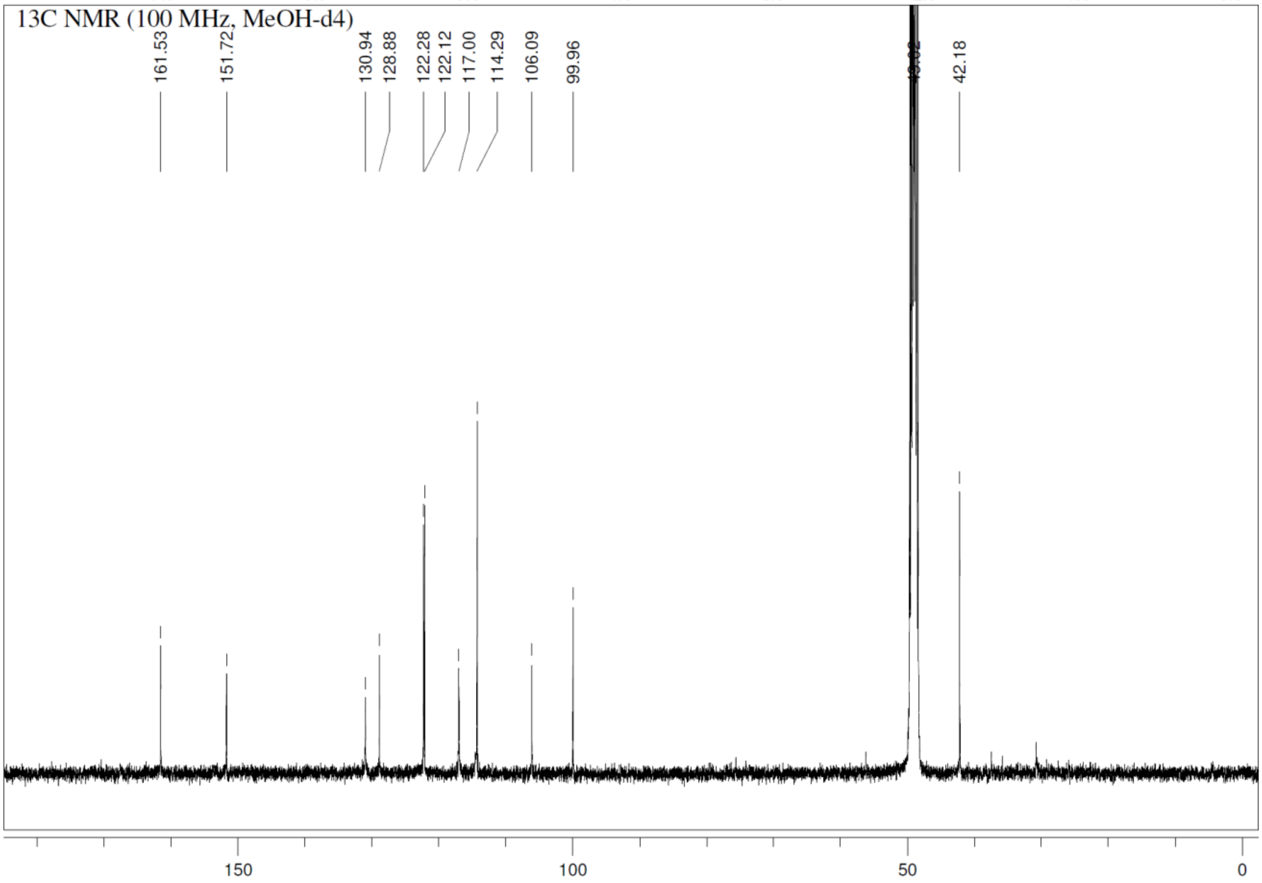

Figure S3. (*E*)-*N*-(3-(2-amino-1*H*-imidazol-4-yl)allyl)-1*H*-indole-2-carboxamide (**2c**).

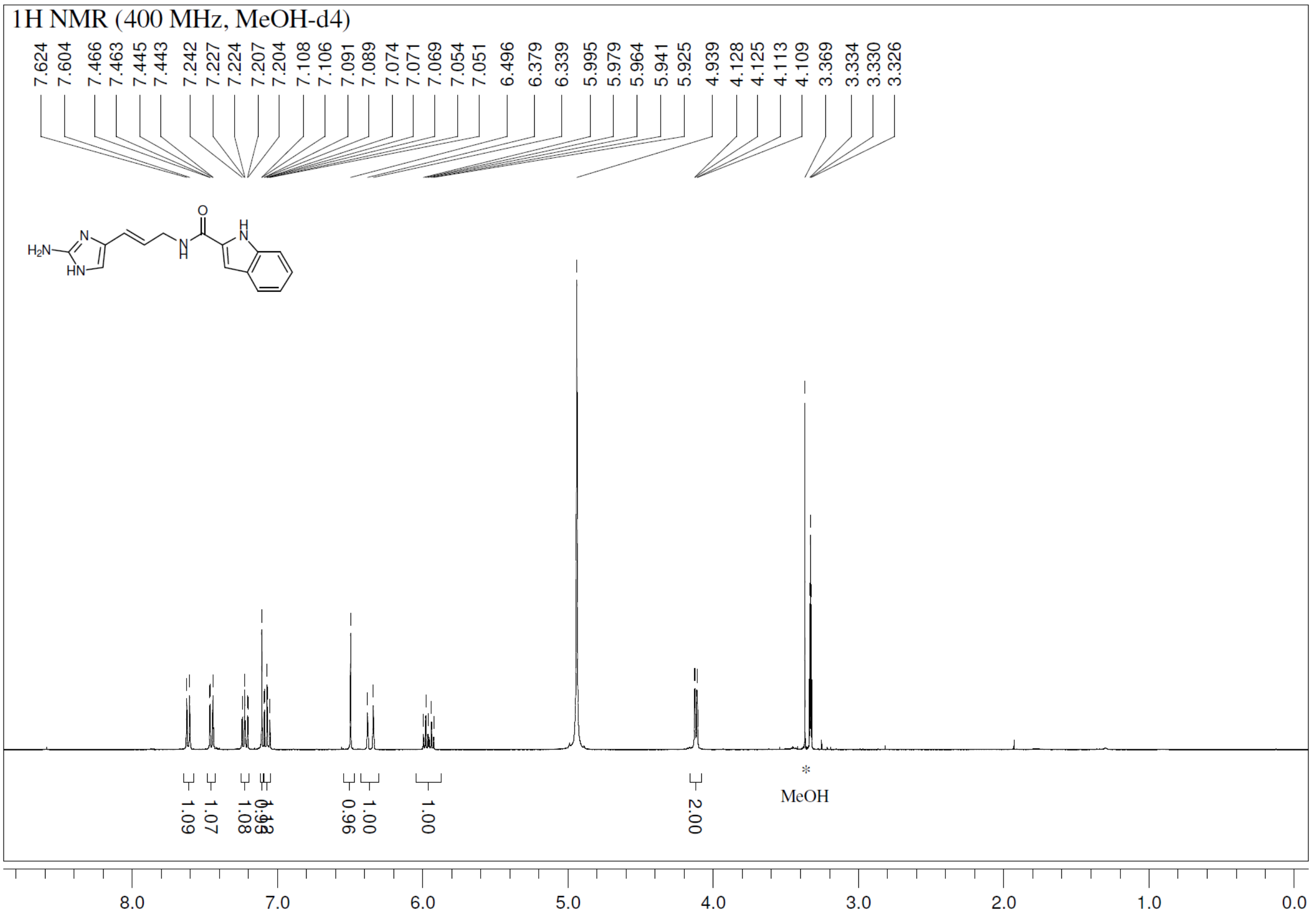

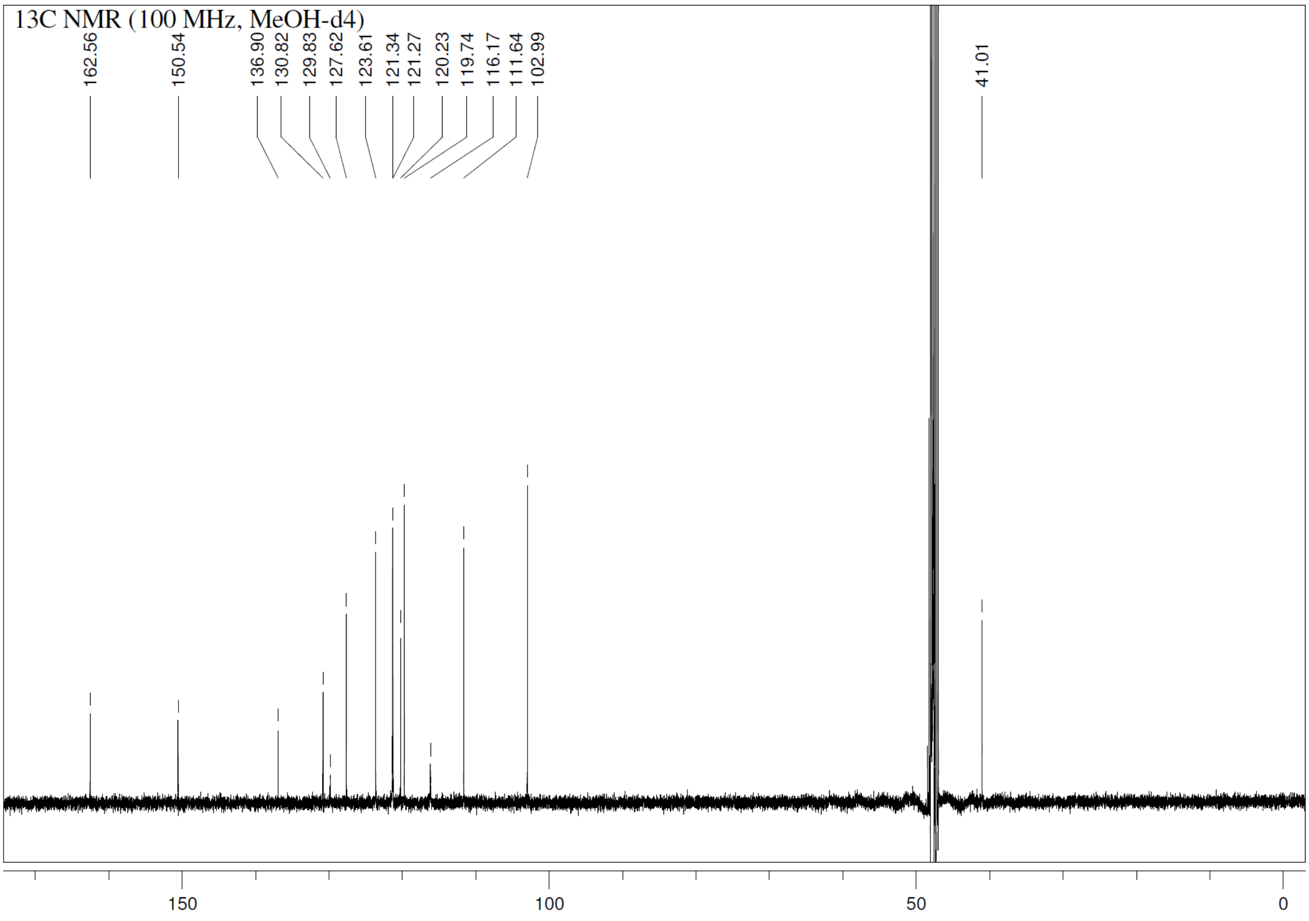

Figure S4. (*E*)-*N*-(3-(2-amino-1*H*-imidazol-4-yl)allyl)-5-fluoro-1*H*-indole-2-carboxamide (**2d**).

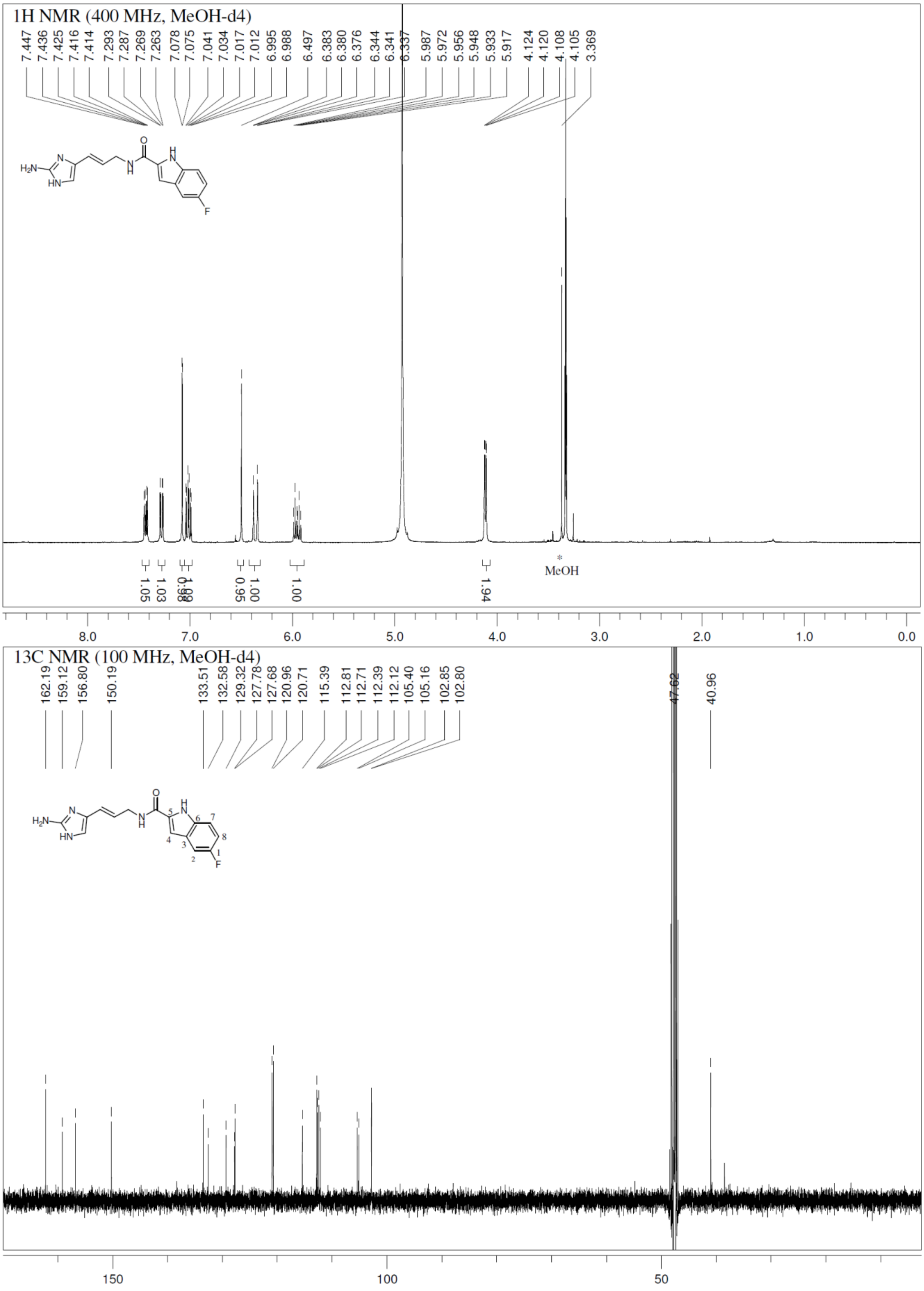

Figure S5. *tert*-Butyl 2-amino-4-(3-(5-methoxy-1*H*-indole-2-carboxamido)phenyl)-1*H*-imidazole-1-carboxylate (**5f**).

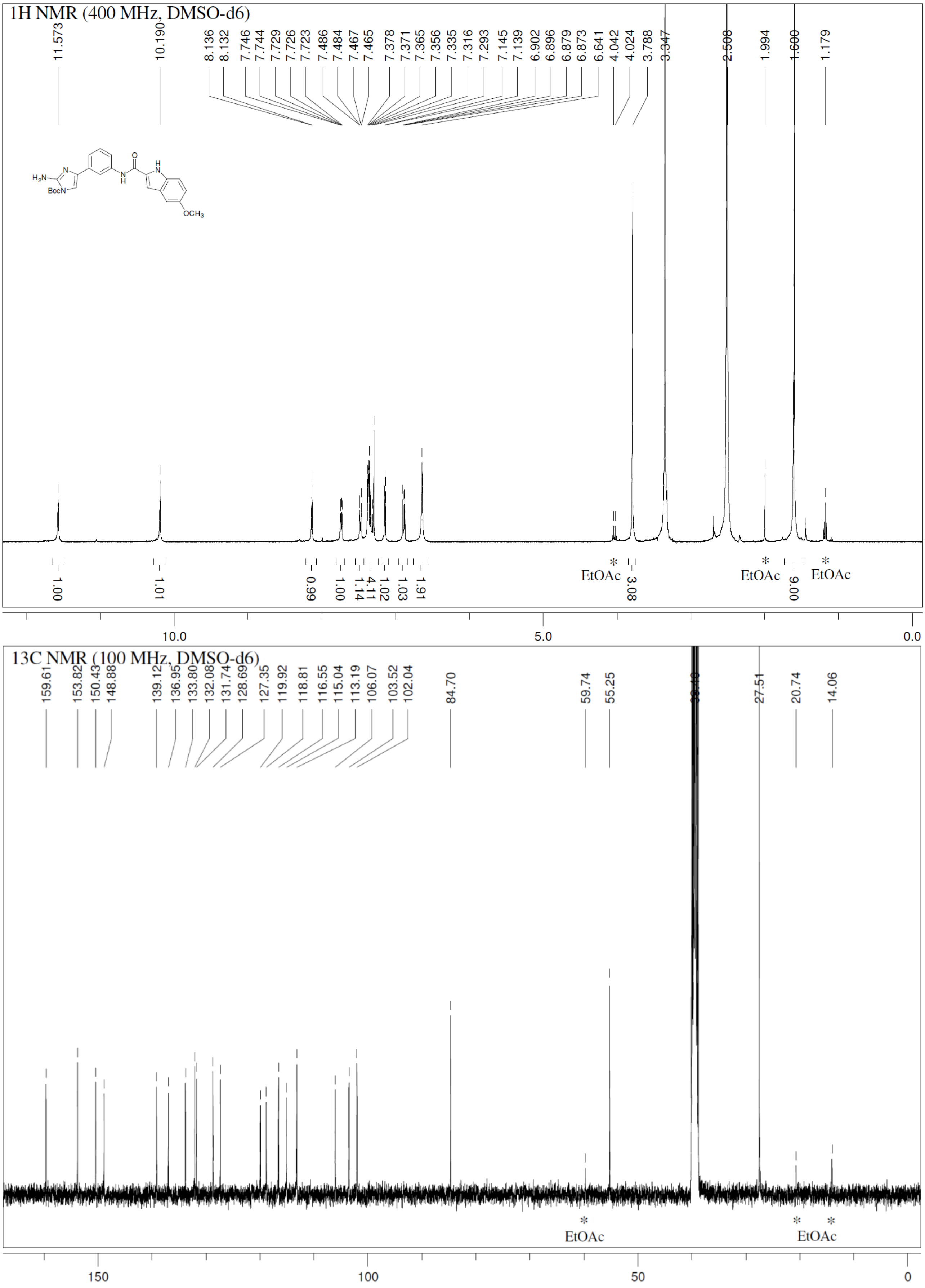

Figure S6. *tert*-Butyl 2-amino-4-(3-(5-(trifluoromethoxy)-1*H*-indole-2-carboxamido) phenyl)-1*H*-imidazole-1-carboxylate (**5g**).

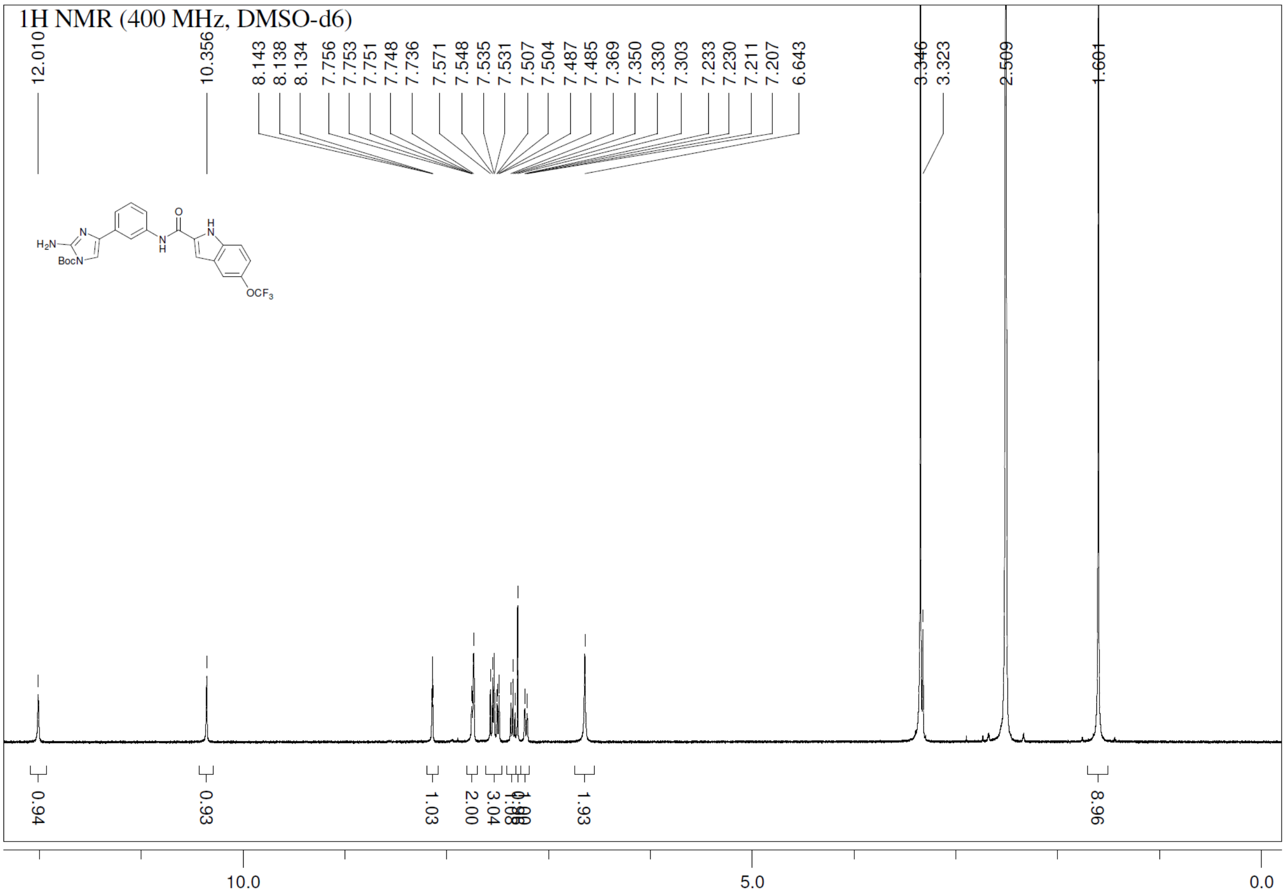

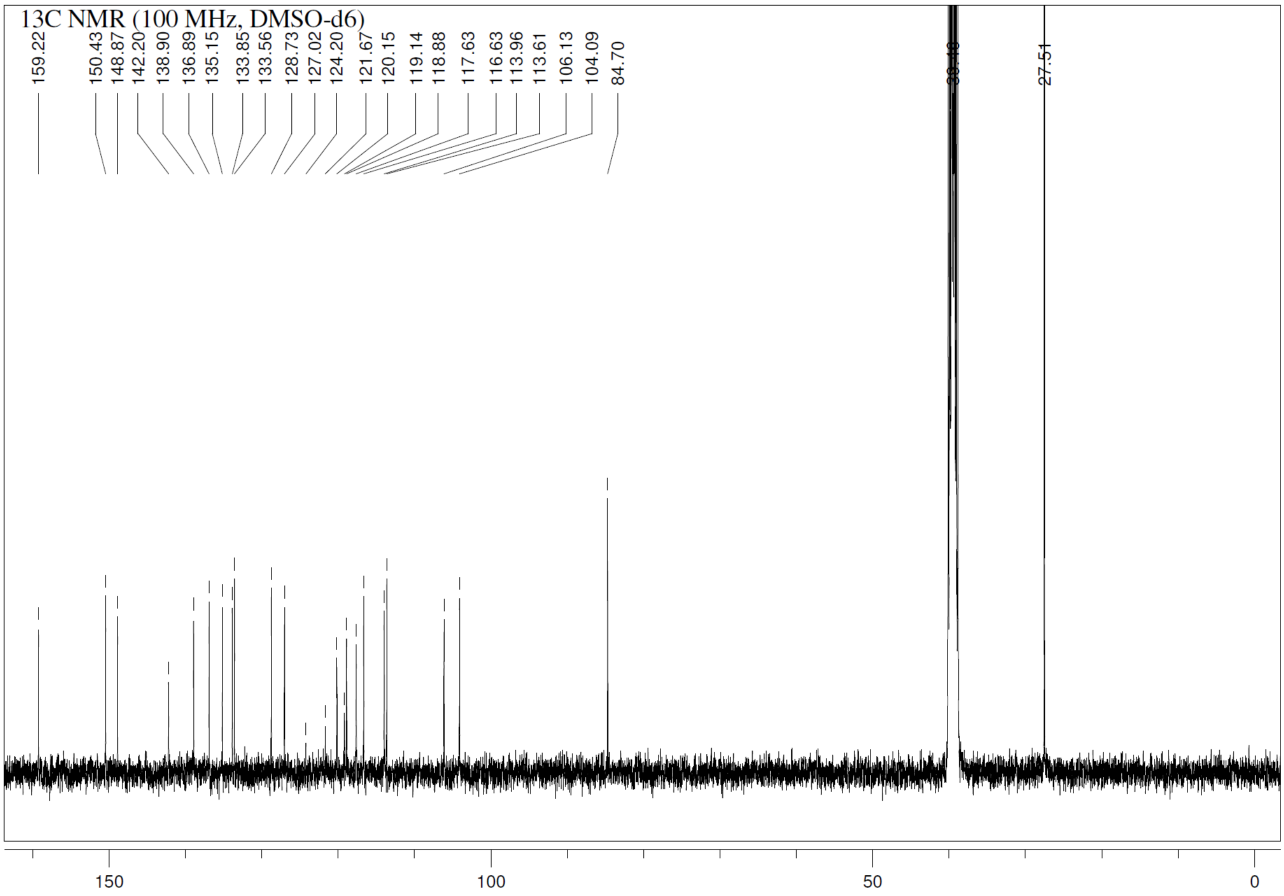

Figure S7. *tert*-Butyl 2-amino-4-(3-(5-(benzyloxy)-1*H*-indole-2-carboxamido)phenyl)-1*H*-imidazole-1-carboxylate (**5h**).

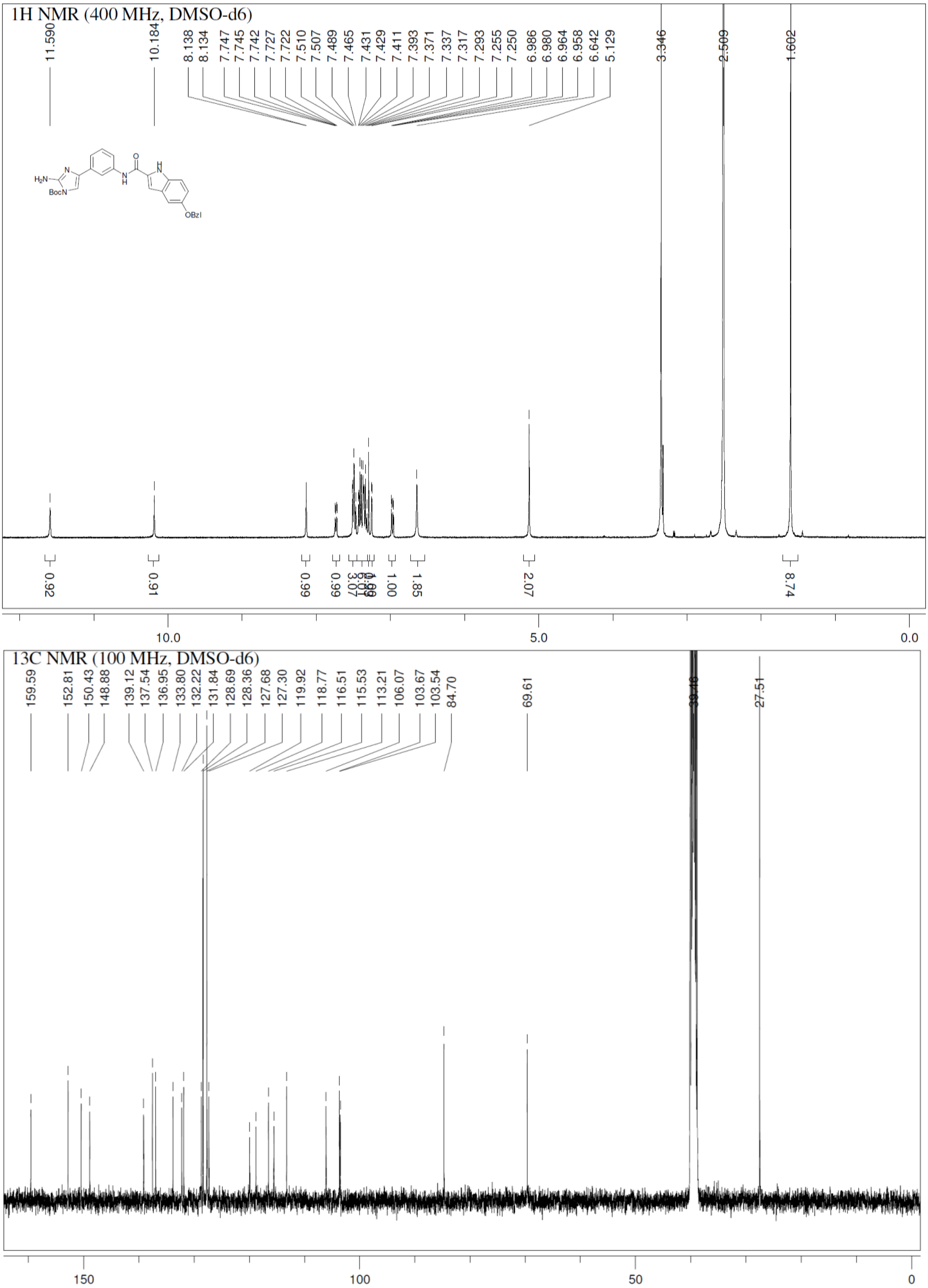

Figure S8. *tert*-Butyl 2-amino-4-(3-(5-chloro-1*H*-indole-2-carboxamido)phenyl)-1*H*-imidazole-1-carboxylate (**5i**).

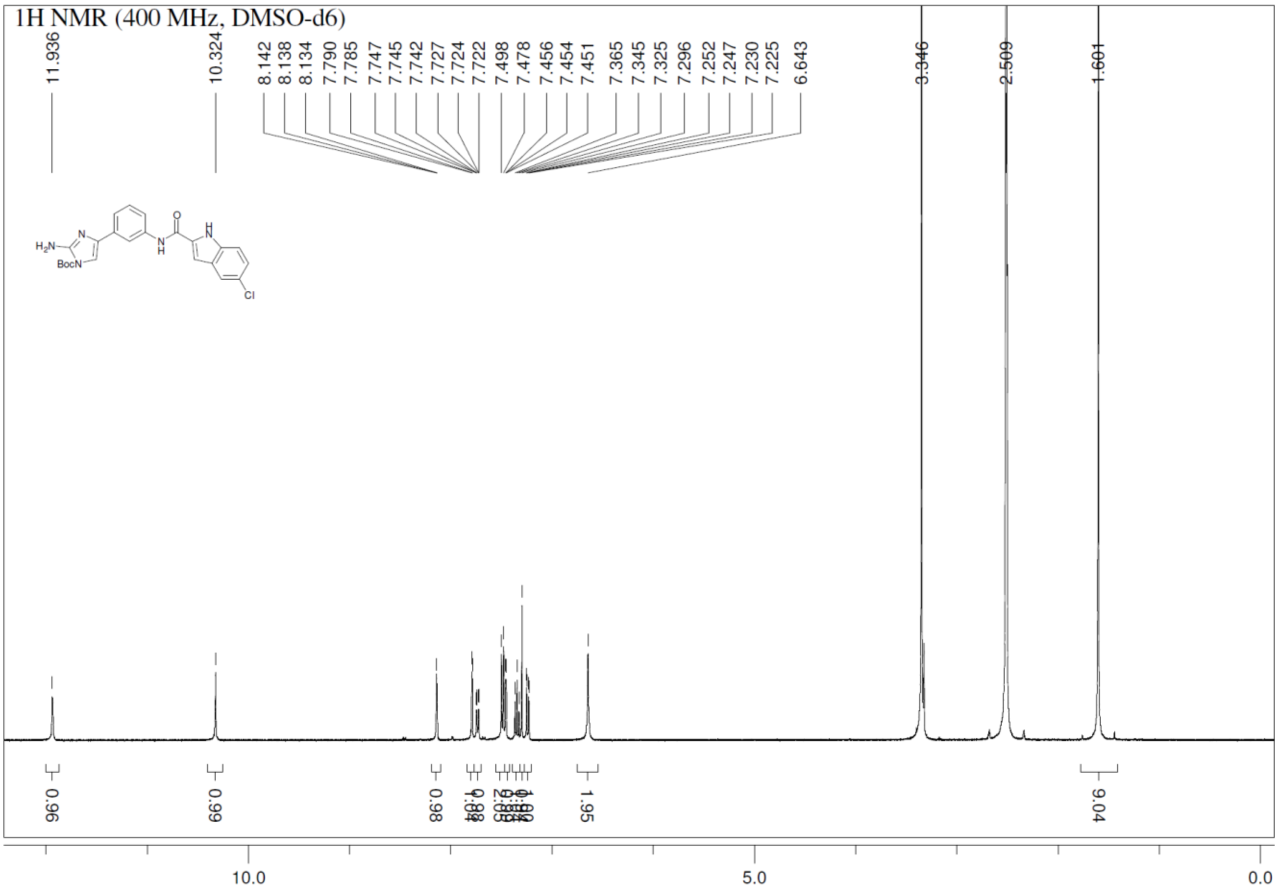

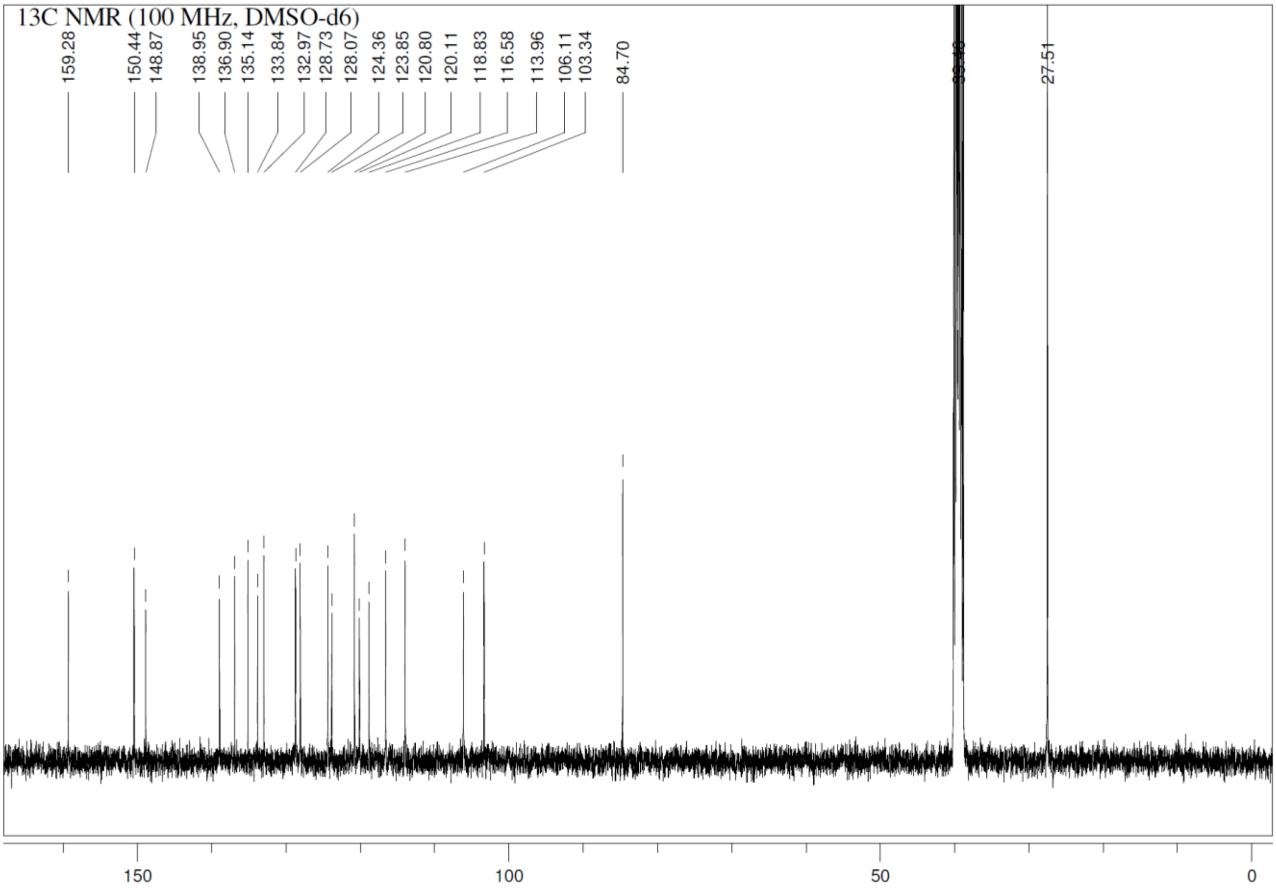

Figure S9. *tert*-Butyl 2-amino-4-(3-(5-fluoro-1*H*-indole-2-carboxamido)phenyl)-1*H*-imidazole-1-carboxylate (**5j**).

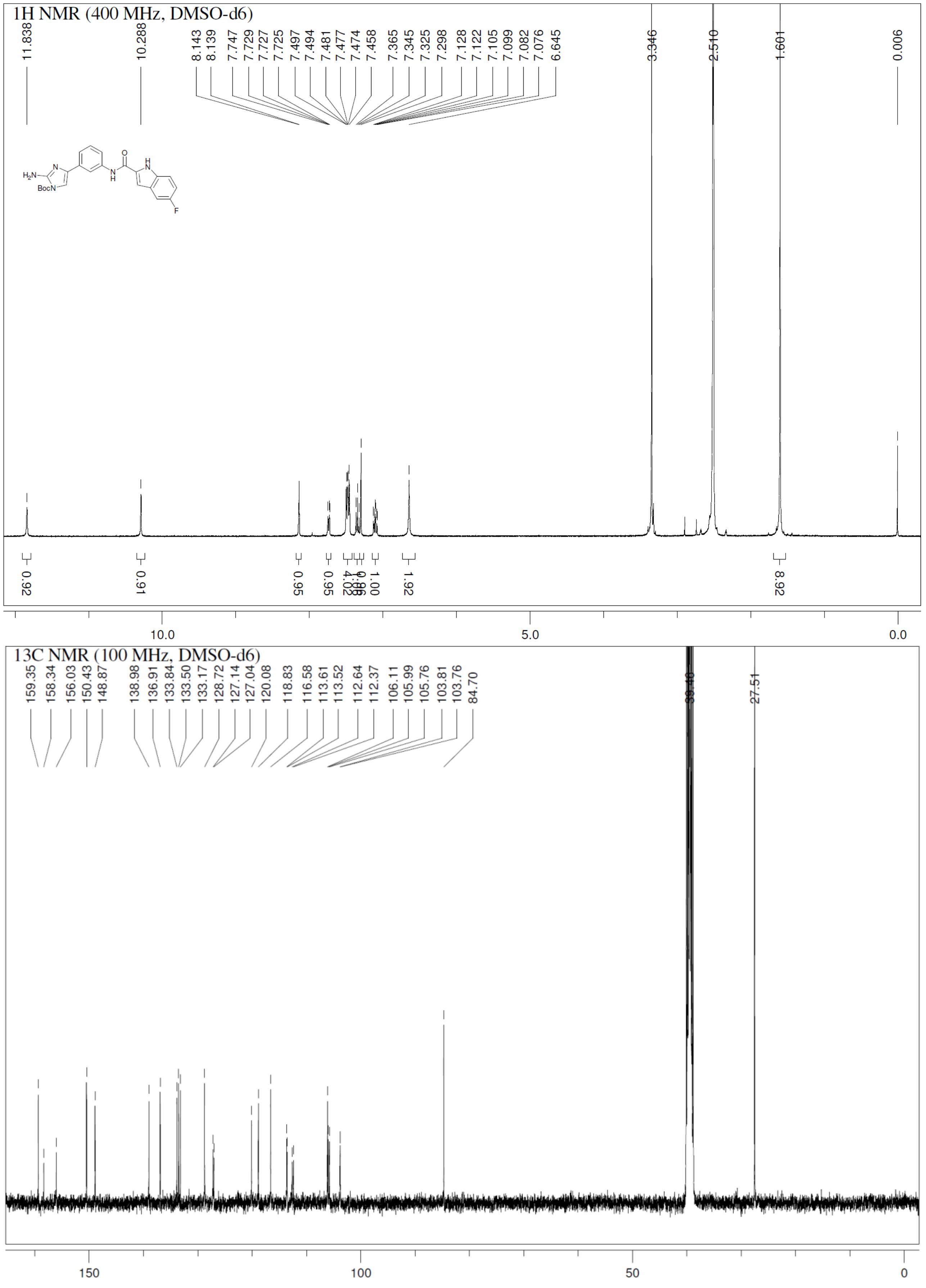

Figure S10. *tert*-Butyl 4-(3-(4*H*-thieno[3,2-*b*]pyrrole-5-carboxamido)phenyl)-2-amino-1 *H*-imidazole-1-carboxylate (**5k**).

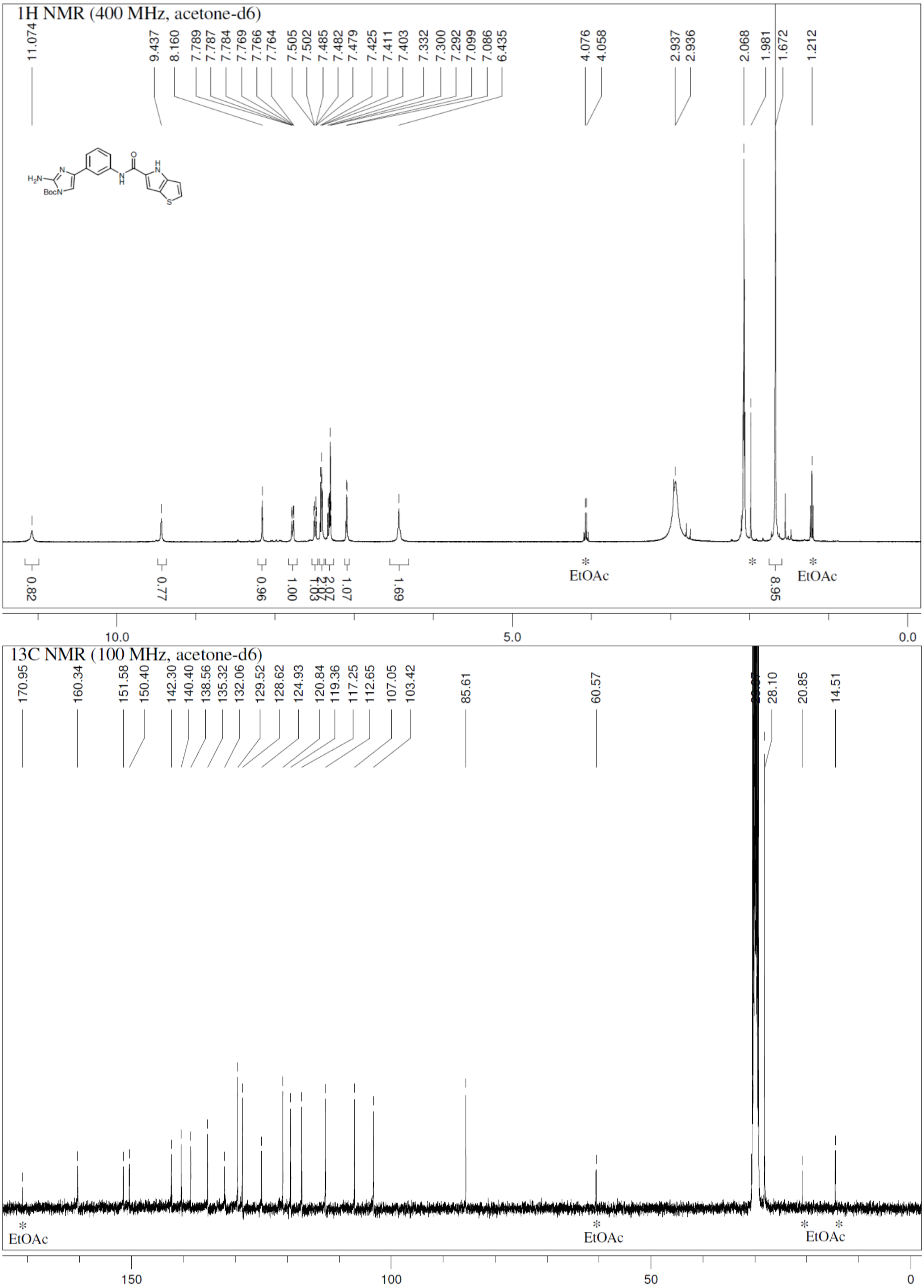

Figure S11. 2-Amino-4-(3-(5-methoxy-1*H*-indole-2-carboxamido)phenyl)-1*H*-imidazol-3-ium chloride (**6f**).

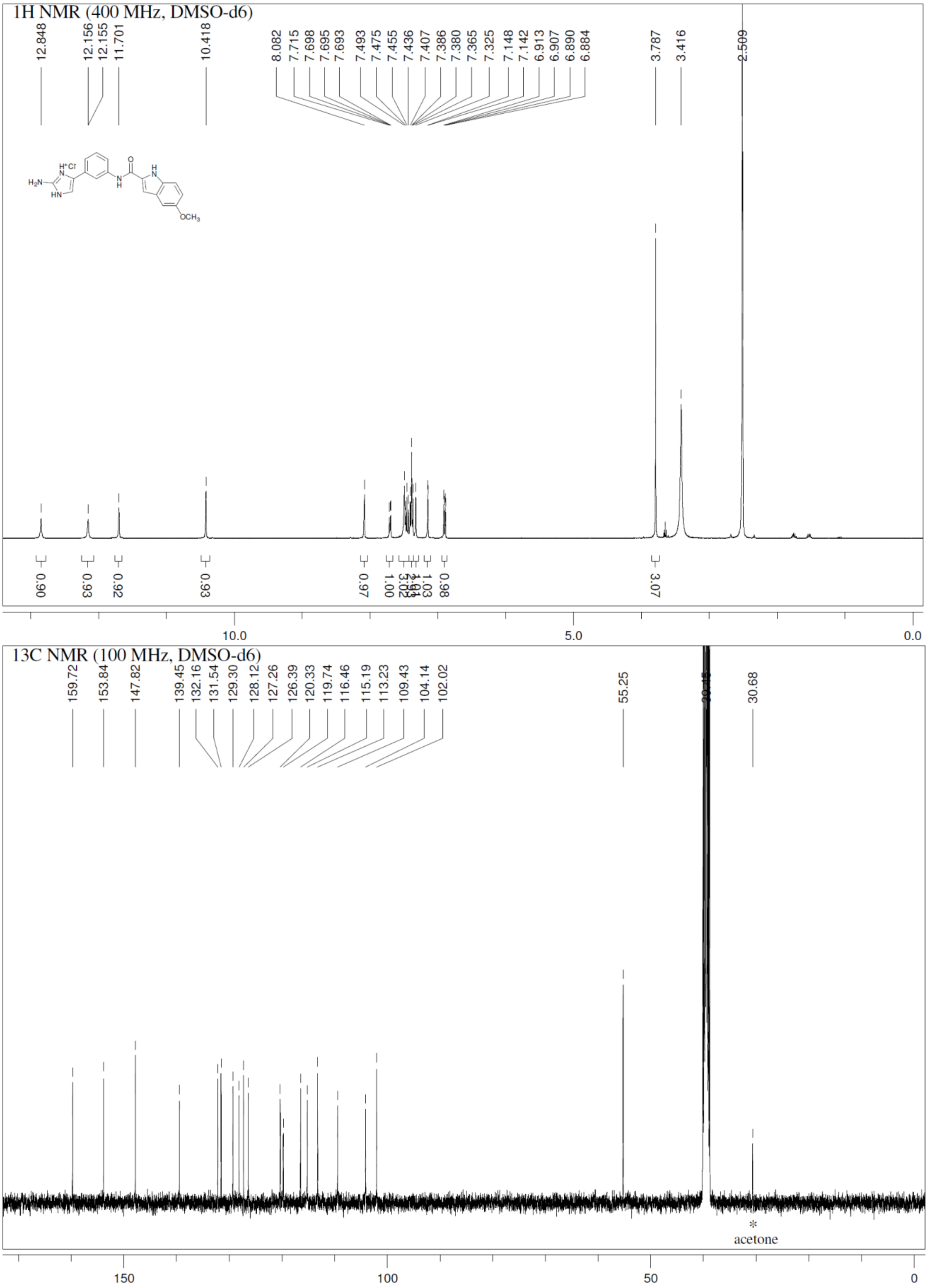

Figure S12. 2-Amino-4-(3-(5-(trifluoromethoxy)-1*H*-indole-2-carboxamido)phenyl)-1*H*-imidazol-3-ium chloride (**6g**).

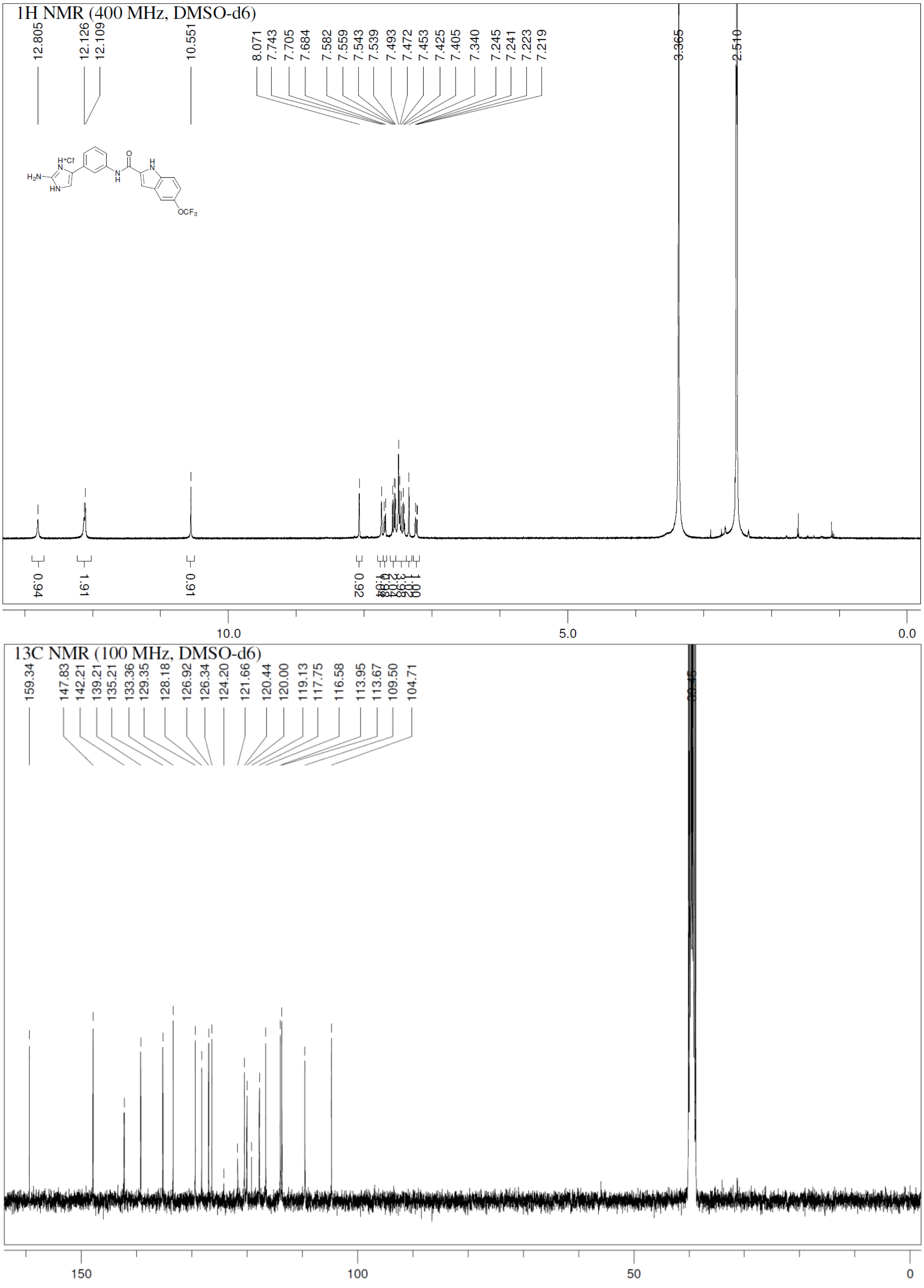

Figure S13. 2-Amino-4-(3-(5-(benzyloxy)-1*H*-indole-2-carboxamido)phenyl)-1*H*-imidazol-3-ium chloride (**6h**).

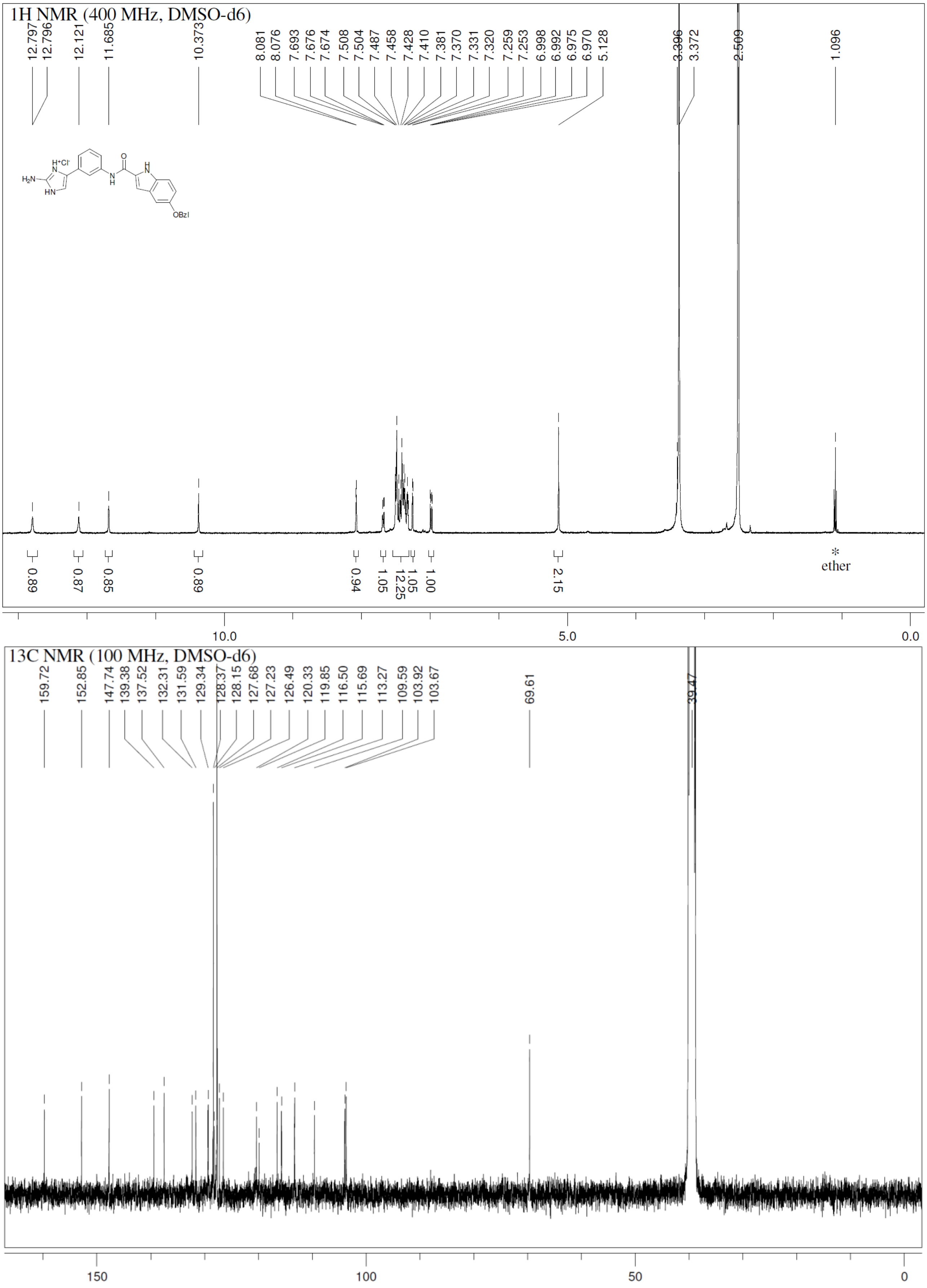

Figure S14. 2-Amino-4-(3-(5-chloro-1*H*-indole-2-carboxamido)phenyl)-1*H*-imidazol-3-ium chloride (**6i**).

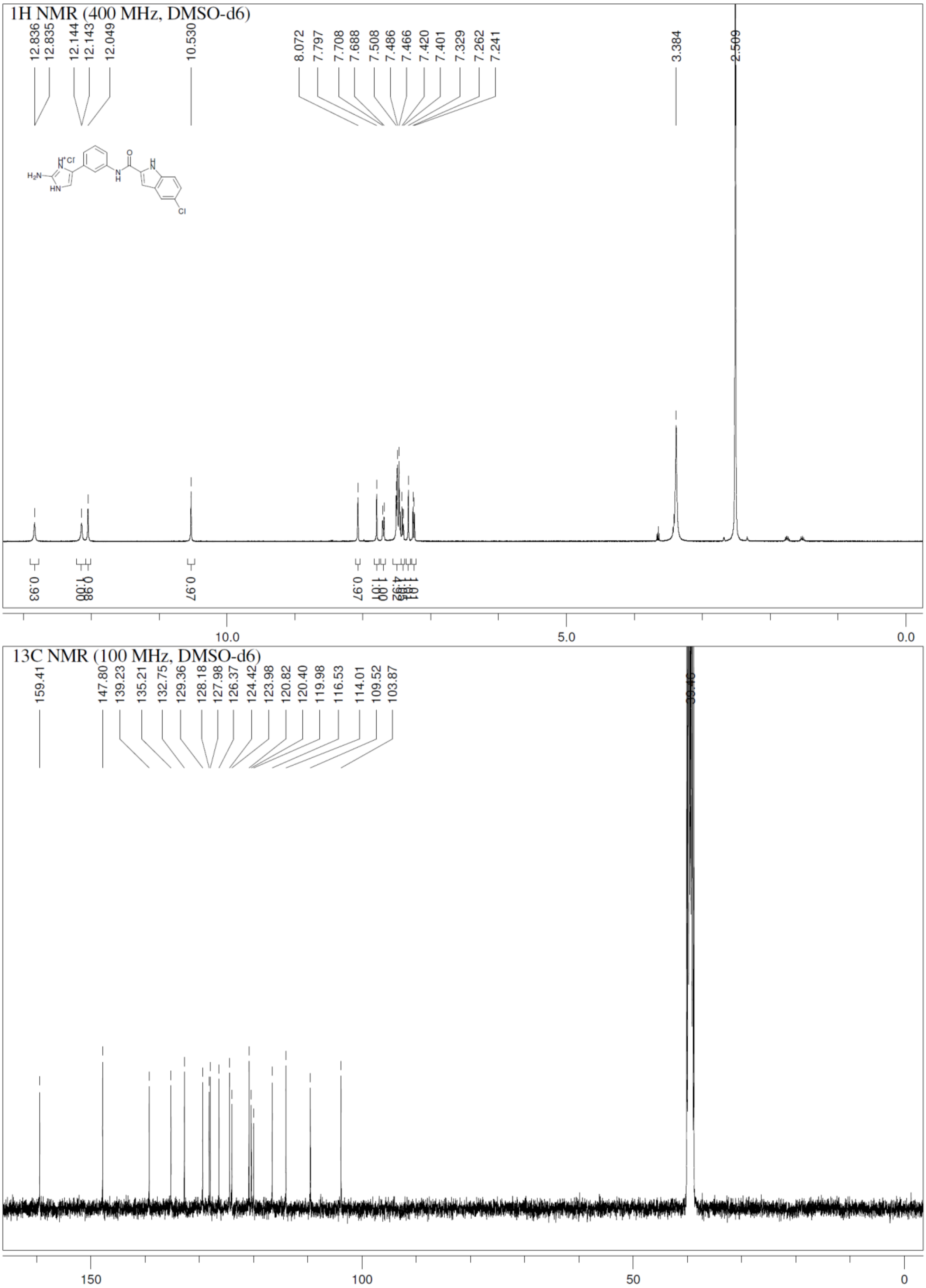

Figure S15. 2-Amino-4-(3-(5-fluoro-1*H*-indole-2-carboxamido)phenyl)-1*H*-imidazol-3-ium chloride (**6j**).

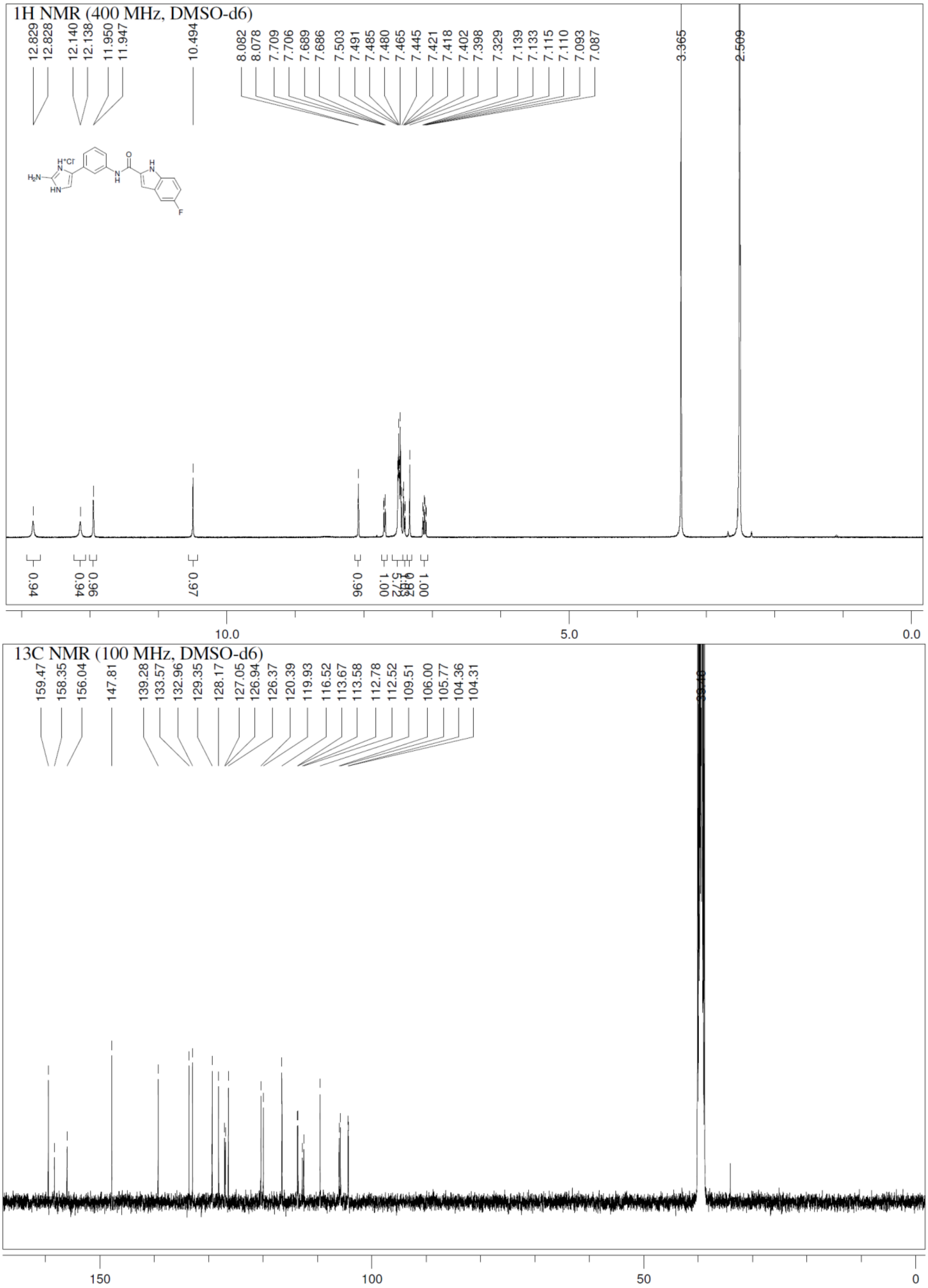

Figure S16. 4-(3-(4*H*-Thieno[3,2-*b*]pyrrole-5-carboxamido)phenyl)-2-amino-1*H*-imidazol-3-ium chloride (**6k**).

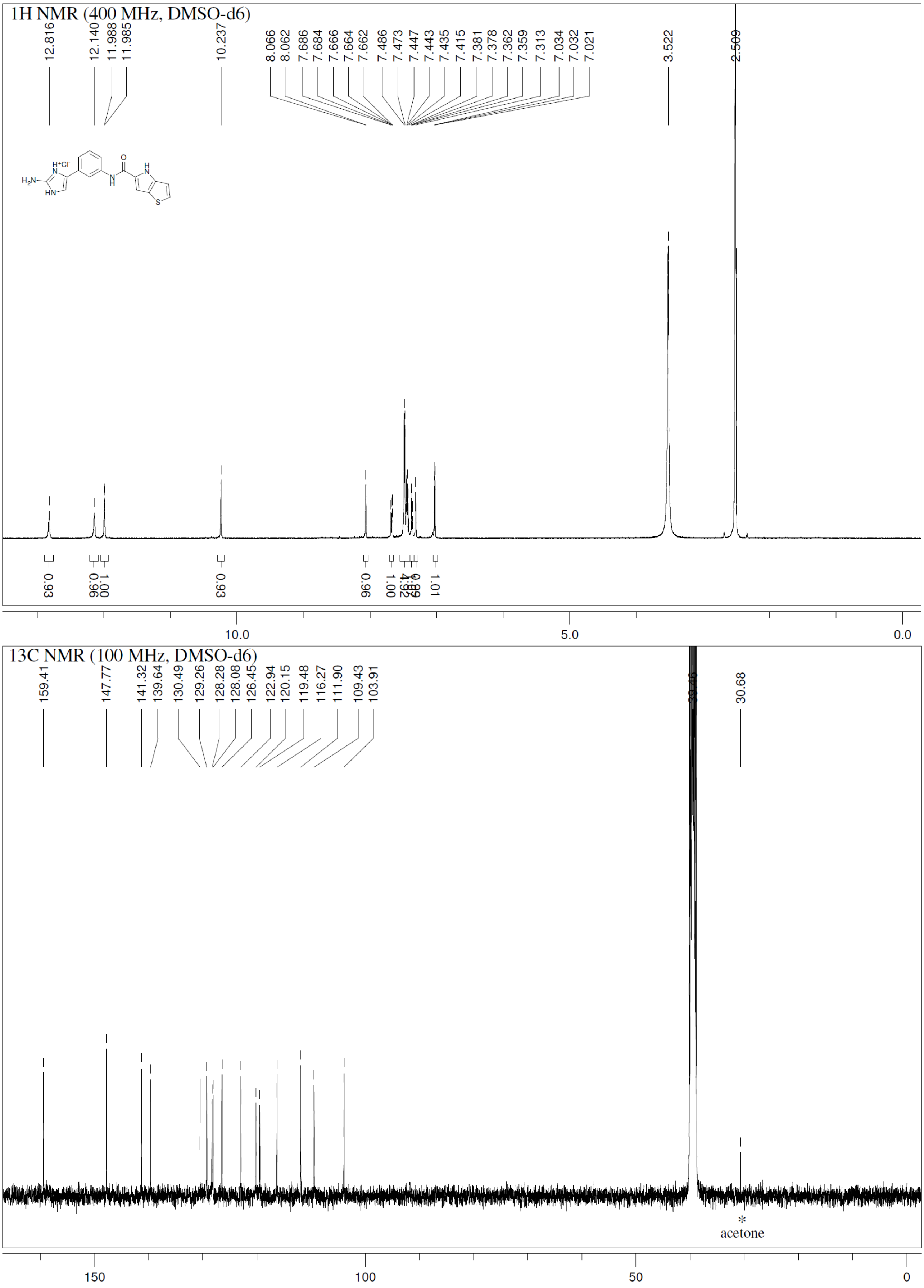

Figure S17. *tert*-Butyl 2-amino-4-(3-(5-hydroxy-1*H*-indole-2-carboxamido)phenyl)-1*H*-imidazole-1-carboxylate (**7**).

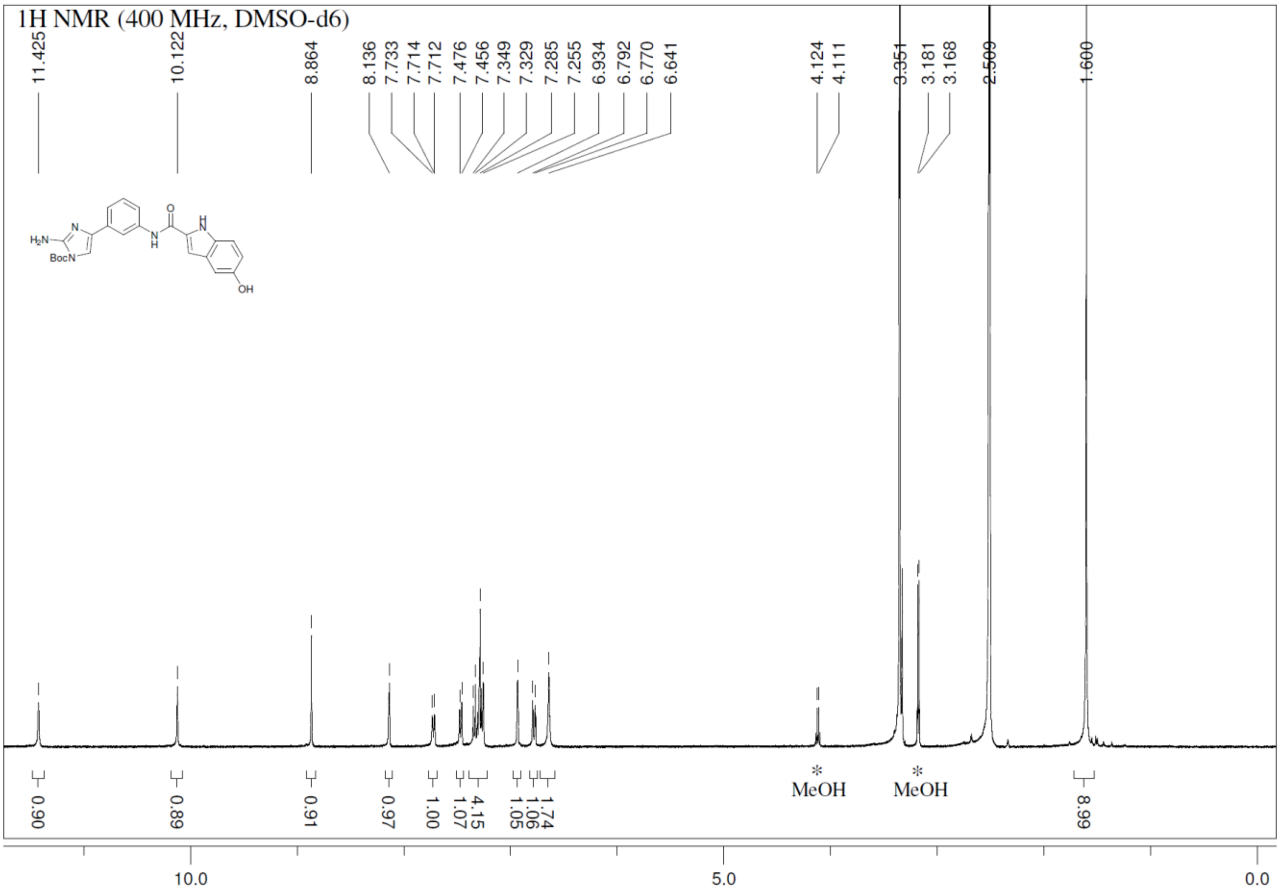

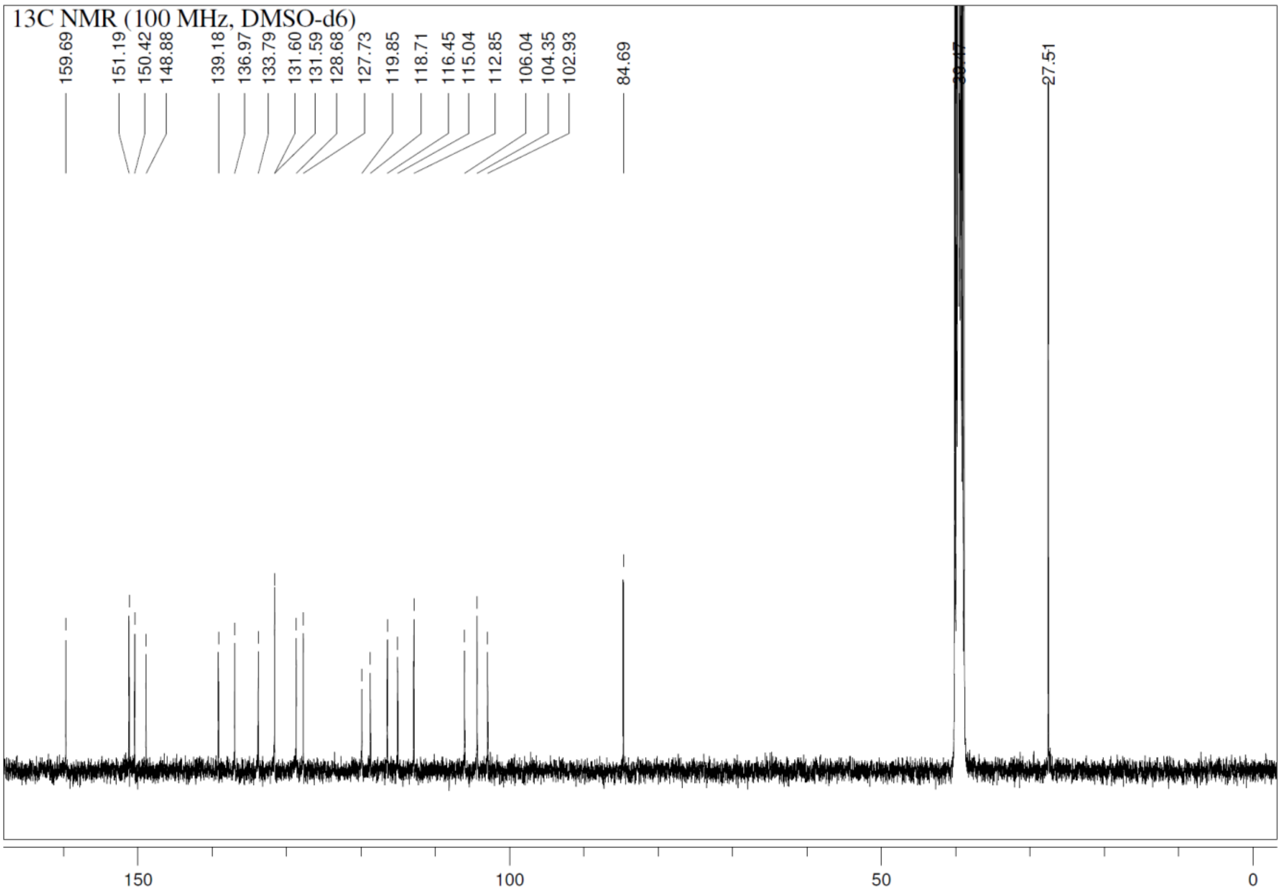

Figure S18. 2-Amino-4-(3-(5-hydroxy-1*H*-indole-2-carboxamido)phenyl)-1*H*-imidazol-3-ium chloride (**8**).

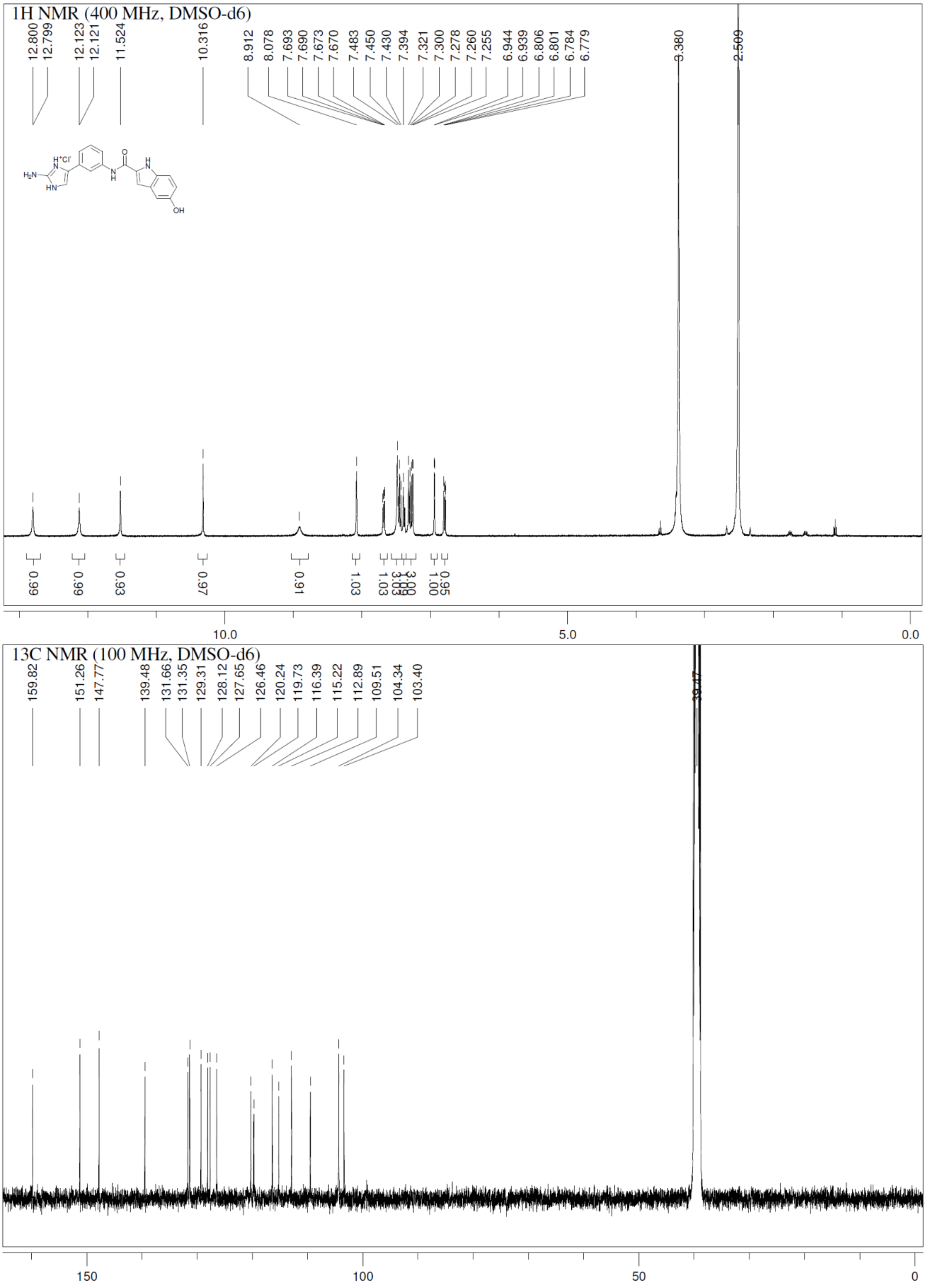

Figure S19. 1-Benzyl-4-(3-nitrophenyl)-1*H*-imidazol-2-amine (**13**).

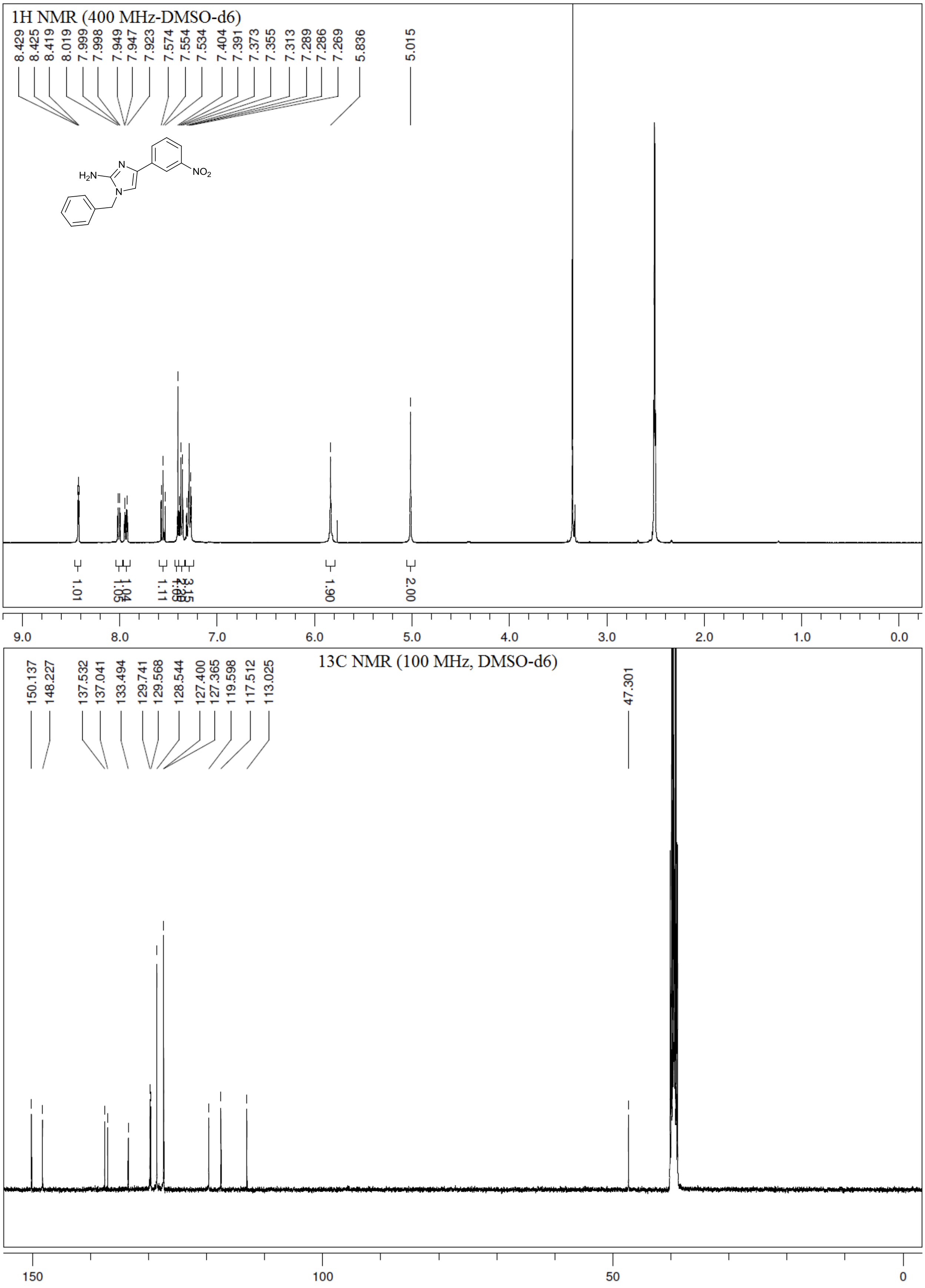

Figure S20. 4-(3-Aminophenyl)-1-benzyl-1*H*-imidazol-2-amine (**14**).

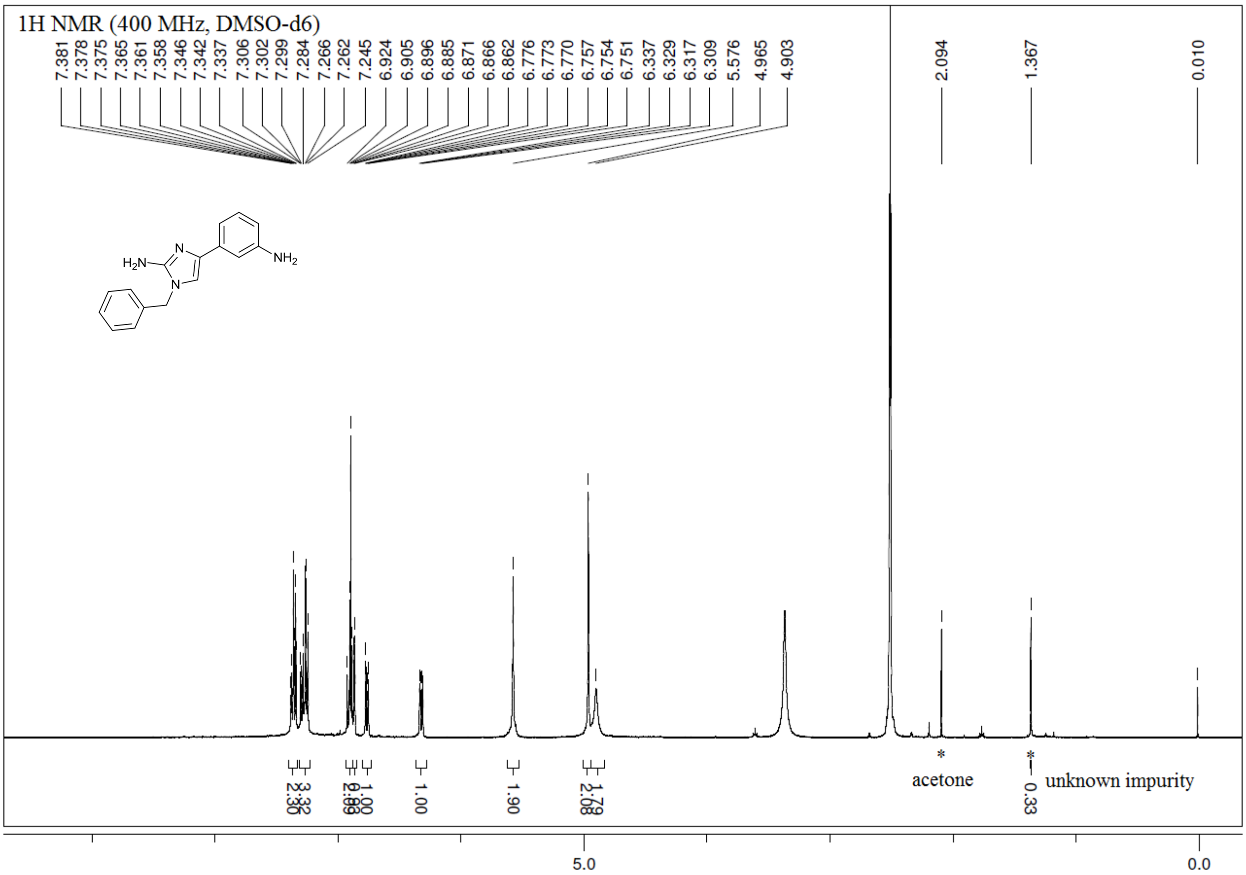

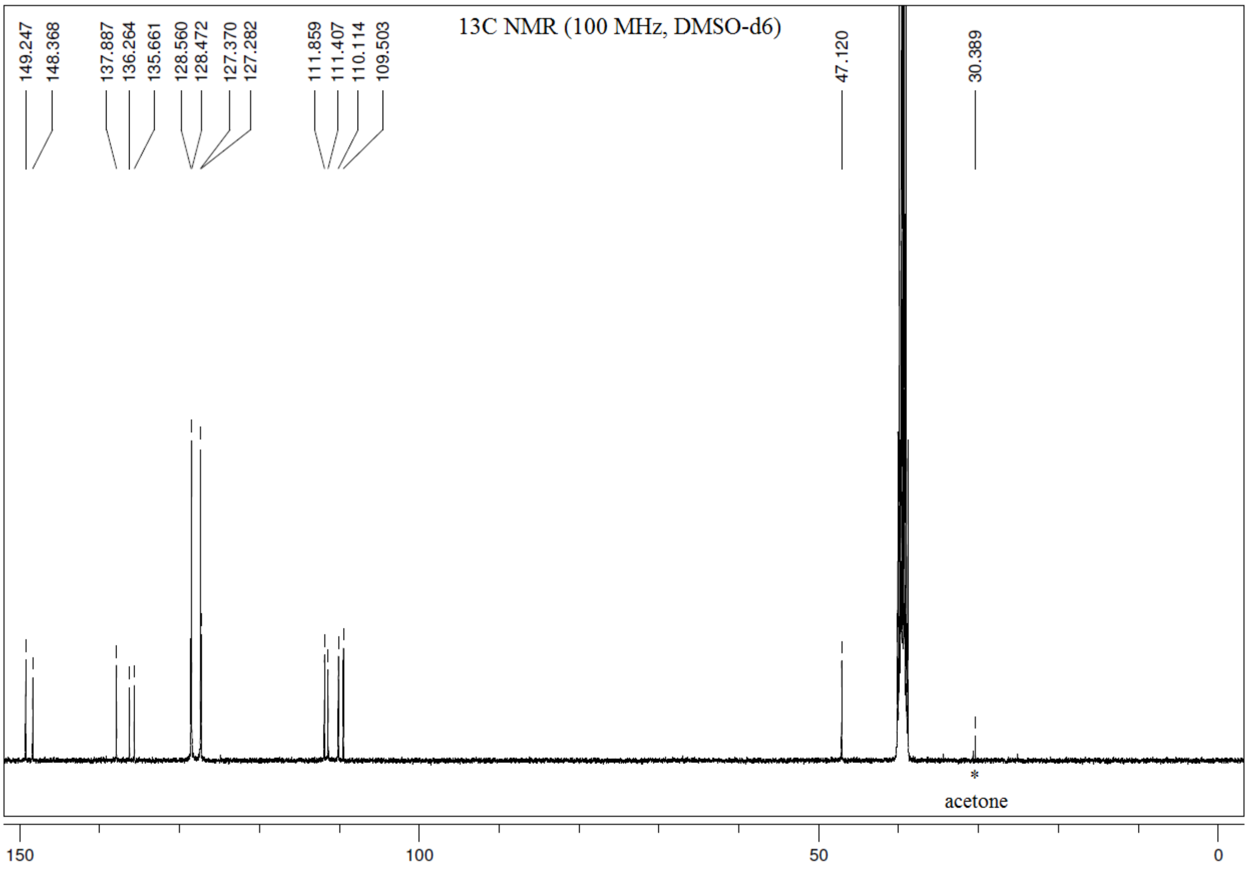

Figure S21. *N*-(3-(2-Amino-1-benzyl-1*H*-imidazol-4-yl)phenyl)-1*H*-pyrrole-2-carboxamide (**15**).

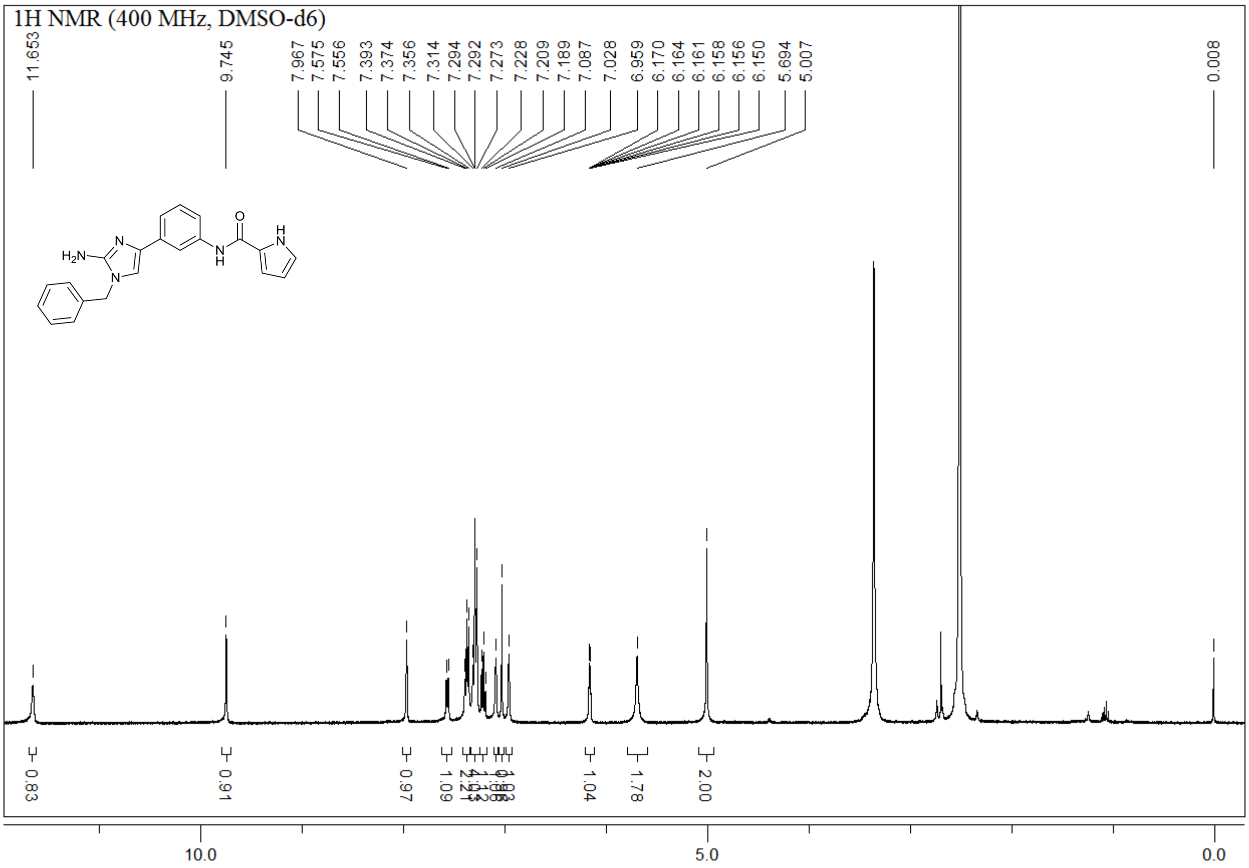

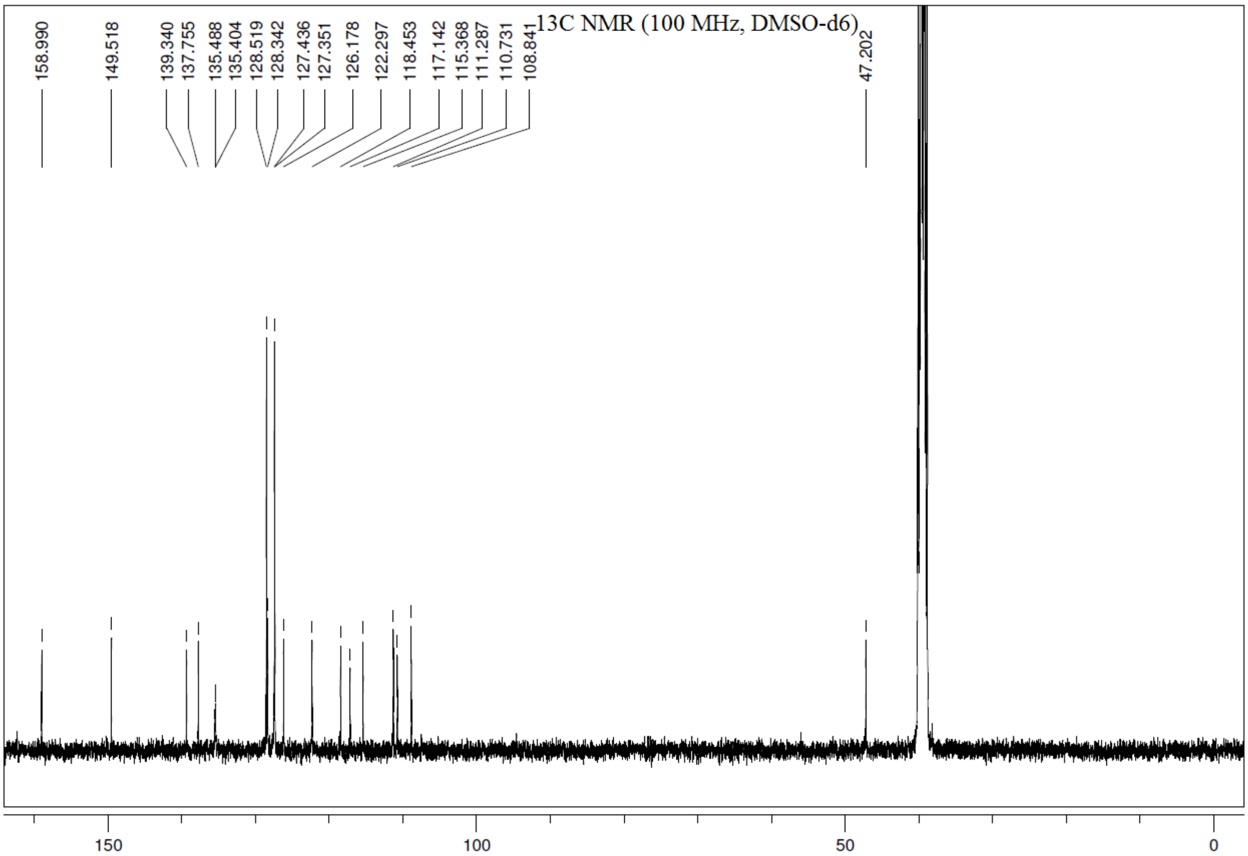

Figure S22. 4-(3-(((1*H*-Pyrrol-2-yl)methyl)amino)phenyl)-1-benzyl-1*H*-imidazol-2-amine (**16**).

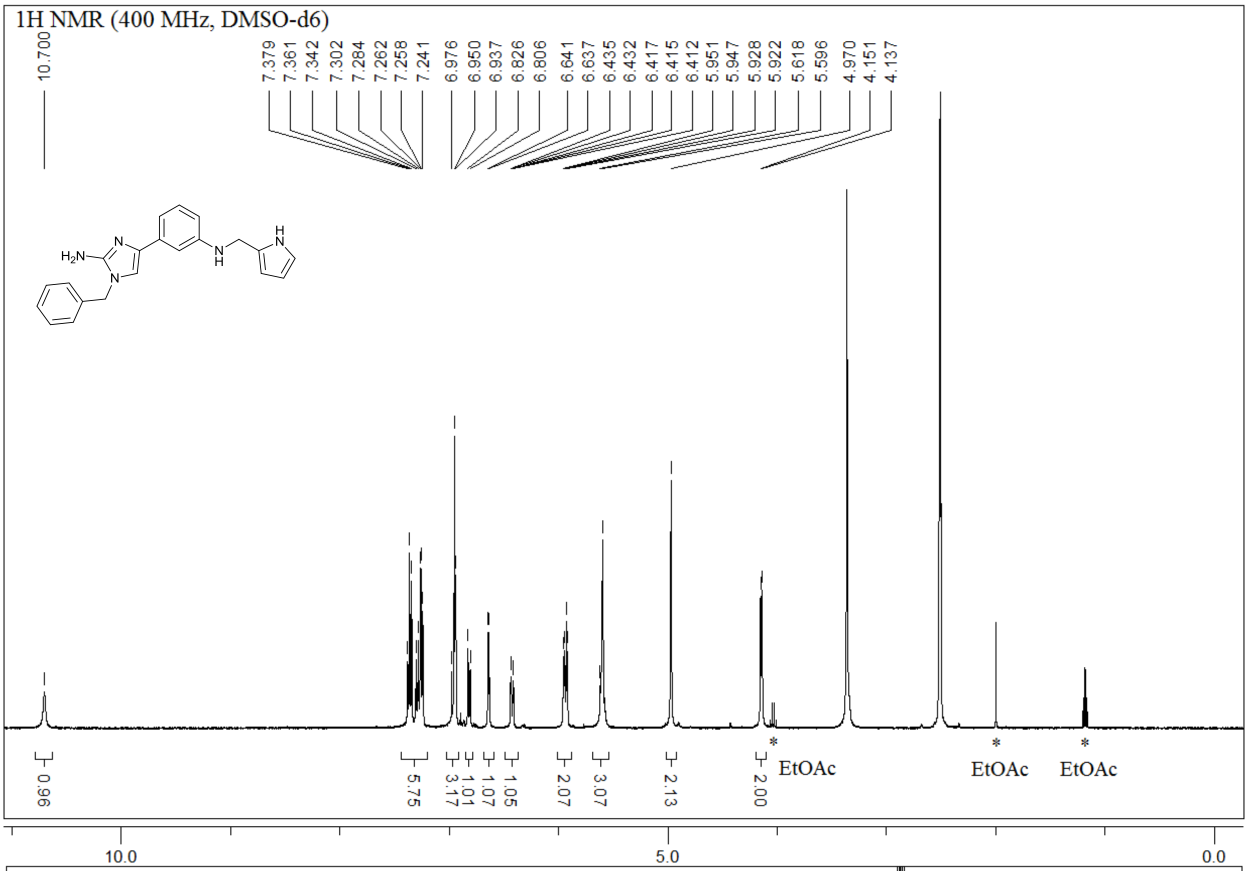

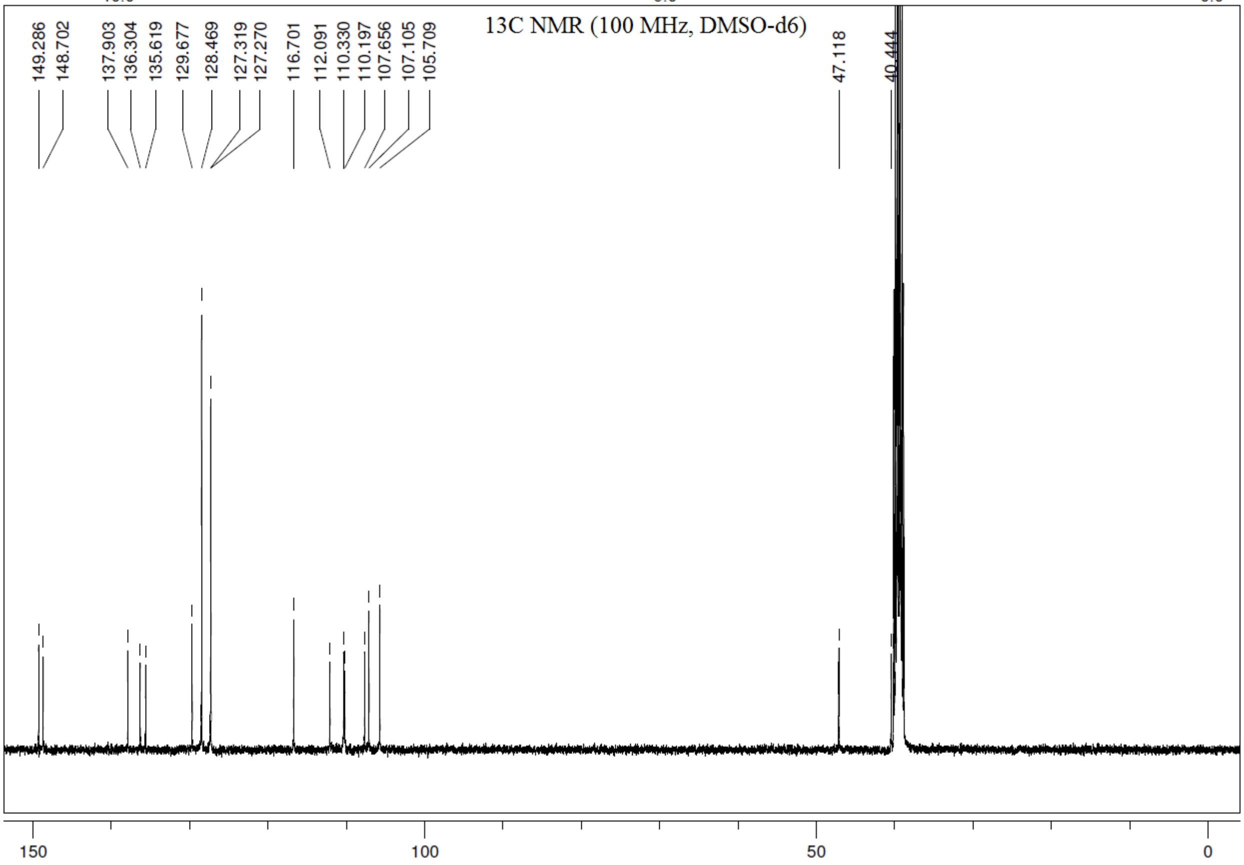

Supplement: Supplementary File 1 — Supplementary Information (PDF, 3708 KB) [file marinedrugs-12-00940-s001.pdf]
